# CycleMix: Gaussian mixture modeling of the cell cycle

**DOI:** 10.1093/bioadv/vbag179

**Published:** 2026-06-22

**Authors:** Jack Edward Peplinski, Tallulah S Andrews

**Affiliations:** Department of Electrical and Computer Engineering & Ivey School of Business, University of Western Ontario, London, ON N6A 3K7, Canada; Department of Biochemistry, Schulich School of Medicine and Dentisrty, University of Western Ontario, London, ON N6A 3K7, Canada; Department of Oncology, Schulich School of Medicine and Dentisrty, University of Western Ontario, London, ON N6A 3K7, Canada; Department of Computer Science, University of Western Ontario, London, ON N6A 3K7, Canada

## Abstract

**Motivation:**

The cell cycle is a crucial component of many biological processes, including cancer, tissue repair, and inflammation. However, due to the heterogeneity of this cycle it has been difficult to assess the extent of proliferation in clinical tissues. Single-cell RNAseq (scRNAseq) and spatial transcriptomics enable high resolution measurement of gene expression enabling the classification of individual cells into their cycling state. However, current methods are limited to classifying cells into only three states: G1, S, G2M and have unproven accuracy on modern datasets.

**Results:**

We show that Seurat and Cyclone the most widely used methods for cell cycle assignment have poor performance on modern droplet-based datasets. In particular, Seurat frequently labels mature non-cycling cells (e.g. neurons) as actively cycling. We present CycleMix, an alternative cell cycle assignment algorithm that can flexibly assign cells into any number of states provided sufficient marker genes as well as being capable of identifying when cells are not cycling. We demonstrate its superior performance for cell cycle assignment and regression of cell cycle expression patterns on six diverse droplet-based scRNAseq datasets.

**Availability and implementation:**

CycleMix is available as an R package on Bioconductor, and on github: https://github.com/tallulandrews/CycleMix.

## 1 Introduction

The cell cycle is a fundamental biological process that enables the survival and reproduction of all living things. Dysregulation of the cell cycle has been implicated in diseases such as cancer (Hanahan and Weinberg 2011, Fares *et al.* 2020), neurodegeneration (Wang *et al.* 2009, Joseph *et al.* 2020), polycystic kidney disease (Lee *et al.* 2021), and schizophrenia (Katsel *et al.* 2008). More recently, cell cycle length has been hypothesized to control cell differentiation (Abou Chakra *et al.* 2021). However, the cell cycle can also be an unwanted confounding variable when working with cells in culture or in highly proliferative tissues (Kolodziejczyk *et al.* 2015, Blasi *et al.* 2017, Szalai *et al.* 2019).

Single-cell RNA sequencing (scRNAseq) enables high-throughput measurement of heterogeneity within individual cells (Kiselev *et al.* 2019, Luecken and Theis 2019, Andrews *et al.* 2021, Heumos *et al.* 2023, Gulati *et al.* 2025). Datasets now routinely include upwards of 100 000 single cells obtained from dozens of individual samples (Zeisel *et al.* 2018, Sikkema *et al.* 2022, Almeida *et al.* 2024, Andrews *et al.* 2024, Cui *et al.* 2024). The cell cycle represents a major source of variability within these datasets, both as variability between proliferative cells in different points in the cell cycle (McDavid *et al.* 2016, Lauridsen *et al.* 2018) as well as variability between proliferative and non-proliferative cells (Gu *et al.* 2024). While variability across the cell cycle is unambiguously a confounder in scRNAseq analysis, it is frequently the case that variability between proliferative and quiescent cells is an important feature of the tissue. For instance, increased and uncontrolled proliferation is a hallmark of cancer and proliferation rate is a predictor of future metastatic disease in some cancer types (Costa *et al.* 1997, Heslin *et al.* 1998, Hanahan and Weinberg 2011, Green *et al.* 2016). In contrast, loss of neuroblast proliferation has been associated with cognitive decline following SARS-infection (Soung *et al.* 2022) and during ageing (Ahlenius *et al.* 2009, Solano Fonseca *et al.* 2016). In the liver, a common consequence of cholestatic fibrosis is an increased proliferation of bile-duct cells and loss of hepatic proliferation and regenerative capacity (El-Araby *et al.* 2021, Owen *et al.* 2022).

Activation of proliferation can be a crucial step in normal and diseased biological processes, for instance T-helper cells have been observed to increase proliferation at the point of cell-fate decision (Lönnberg *et al.* 2017). In cancer, the activation, dysregulation, and suppression of proliferation affect drug persistence (Pu *et al.* 2023), progression and invasion (Deshpande *et al.* 2005), and successful metastasis (Fares *et al.* 2020). Thus, the cell cycle was one of the first biological systems targeted with single-cell analysis using synchronized embryonic stem cells (ESCs) (Sasagawa *et al.* 2013, Buettner *et al.* 2015), fluorescent labelling of key cell cycle markers (Leng *et al.* 2015), and flow cytometry properties (McDavid *et al.* 2014).

In parallel, multiple statistical approaches have been developed to classify single cells into their cell cycle phase, which can be divided into two main approaches: continuous cycle inference and discrete cell classification. Oscope (Leng *et al.* 2015) identified oscillating genes using a 2D sine-wave model which are then used to order cells along the cycle. DeepCycle (Riba *et al.* 2022) combined inferred gene expression dynamics from RNA velocity with angular pseudotime to infer an ordering of cells through the cell cycle. Both are examples of continuous cycle inference methods, which require prior knowledge of which cells are actively cycling and which are quiescent. In contrast, classification based methods such as Cyclone (Scialdone *et al.* 2015), which uses relative expression of gene pairs, and Seurat (Butler *et al.* 2018), which uses average normalized expression of marker genes, assign to each cell a specific phase: G1, S, G2/M. These methods do not explicitly allow cells to be classified as non-cycling, but typically cells in the “G1” phase are assumed to primarily represent non-cycling cells, rather than specifically proliferating cells within the G1 phase. This is particularly true of Seurat which does not use any markers to define the G1 phase but simply classifies all non-S and non-G2M cells as G1.

We present a novel cell cycle classification method, CycleMix, which uses Gaussian mixture models (GMMs) to determine which cells in a single-cell experiment are actively cycling and which stage those cells are currently in. We assess the performance of CycleMix on low-throughput scRNAseq datasets where cell cycle phase was identified using orthogonal experimental approaches, and on high-throughput scRNAseq datasets where *a priori* biological knowledge and author annotations can be used to identify proliferative and quiescent cell types. We show that CycleMix is highly scalable, performs competitively on low-throughput datasets, and is the best performing method on high-throughput datasets compared to current state-of-the-art methods.

## 2 Methods

### 2.1 Classifying cells with CycleMix

Gene signatures for each phase were obtained from the published literature, including Whitfield *et al.* (2002), Tirosh *et al.* (2016a), Macosko *et al.* (2015), and from the Seurat(v4) package (Satija *et al.* 2015). Quiescence/G0 signatures were obtained from Reactome’s “G0” gene list, and Cheung and Jia (2013). Genes upregulated in the specific phase were assigned a weight of +1, while genes downregulated in a specific phase were assigned a weight of −1.

Normalized gene expression values are extracted from a provided SingleCellExperiment or Seurat object, and the weighted average expression across genes is calculated for each phase for each cell. For each phase, a GMM is fit to the resulting cell-specific scores (relying on the central limit theorem). We consider mixtures of 1, 2, or 3 Gaussians and the optimal model is identified using the Bayesian information criterion (BIC). Since any population of cycling cells will have cells in at least two phases: S & G2M, if the BIC identifies a single Gaussian as the best fit model, then no cells are assigned to that phase. Otherwise, cells are assigned to this phase if they were most likely to originate from the Gaussian with the highest mean.

As this approach can lead to a single cell being assigned to multiple phases, the user has the choice to keep all assigned phases or as we do in this article assign cells to the phase with the highest score after rescaling (*Z*-score) each set of phase scores. If a cell is not assigned to any phase, it is labelled as “None”.

#### 2.1.1 Checking fit

To ensure the GMM fits the calculated aggregate scores for each phase, we randomly sampled an equal number of cells from each fitted Gaussian distribution as the observed data. Randomly sampled data were compared to the original observed data using a Kologorov–Smirnov (KS) test. Due to the large size of our scRNAseq datasets (>50 000 cells), the KS test was over-powered, thus we use the *D*-statistic which measures the maximum discrepancy between the two cumulative distribution functions instead. The average *D*-statistic over 100 bootstrap samples was calculated to confirm the quality of the mixture model fit.

#### 2.1.2 Smoothing cell cycle classification

To improve the accuracy of CycleMix cell cycle phase assignments, we provide two options to smooth classifications across similar cells. Firstly, is to use the *k*-nearest neighbors of each cell and assigning the selected cell to the most common phase to amongst its 20 nearest neighboring cells in PCA space. The second is to use a set of clusters, or in our case cell type annotations and assign each group of cells to the most frequent cell cycle phase amongst cells within that group. In order to increase the sensitivity of CycleMix, we additionally set a threshold to assign a cell to a cycling phase (i.e. G1S or G2M) versus non-cycling (i.e. None) of 25% of neighboring cells or cells within a cell type being cycling cells. This threshold was determined empirically based on the observed rates of cycling/non-cycling cells in our quiescent and proliferating cell types and should be tuned to the user’s specific dataset.

#### 2.1.3 Regressing cell cycle phase

CycleMix provides the option to regress out one or more phases of the cell cycle, either using discrete classifications or the quantitative scores for each phase. For discrete classifications, one phase is chosen as the reference stage, and differences between this stage and each other specified stage are calculated using negative binomial regression and this difference is removed. Alternatively, cell cycle differences can be removed by regressing out the scores assigned to each cell for each cell cycle phase. This is calculated using negative binomial regression for the specified phases. In both cases, the regression coefficients are adjusted to account for the number of 0 present in the raw gene expression data, negative expression is not biologically meaningful, as below:


(1) $$p_{i}=\frac{1}{n}\sum_{j}\delta_{ij}$$

where $\delta_{ij}$ is 1 when the expression of gene *i* in cell *j* is greater than 0, and *n* is the total number of cells in that particular phase


(2) $${\hat{C}}_{i}=\frac{C_{i}}{p_{i}}$$

where ${\hat{C}}_{i}$ is correction applied to the normalized expression level for gene *i*, and $C_{i}$ is the original correction calculated from the coefficients from the model.

### 2.2 Benchmarking

#### 2.2.1 Other cell cycle classifiers

Cyclone (Scialdone *et al.* 2015), from scran (v1.22.1), was run on raw counts using the in-built lists of mouse or human cell cycle dependent gene pairs. Seurat (v4.4.0) was run on log-normalized counts per million rescaled such that each cell had total counts equal to 10 000 using identical marker genes as were used for CycleMix.

#### 2.2.2 Datasets

Publicly available datasets with orthogonally determined cell cycle phase were obtained from appropriate databases (Table 1).

**Table 1:** Gold-standard benchmarking datasets.

Dataset	Description	Accession
**Sasagawa *et al.* (2013)**	56 synchronized mouse ES cells by Quartz-seq	GSE42268
**Chen *et al.* (2018)**	Synchronized mouse spermatocytes by Smartseq2	GSE107644
**Buettner *et al.* (2015)**	Cynchronized mouse ES cells by Fluidigm C1	E-MTAB-2805
**McDavid *et al.* (2014)**	Flow sorted human cell-lines by Nanostring nCounter targeted scRNAseq	Publication
**Leng *et al.* (2015)**	FUCCI H1 hESCs by Fluidigm C1	GSE64016

#### 2.2.3 Timing and scalability

All methods were run on a HPC cluster with 1 CPU (Intel Gold 6148 Skylake 2.4 GHz) and 16 Gb of RAM. Algorithm timing included only the cell cycle phase assignment, all data preprocessing steps were excluded. To test scalability, the Buettner dataset was resampled as described below generating increasing numbers of cells.

#### 2.2.4 Resampling data

For Chen, Buettner, and Leng datasets, we generated synthetic data more similar to current state-of-the-art droplet-based scRNAseq. We obtained estimates of total UMIs/cell from 10 000 cells from two recent scRNAseq datasets generated using the 10X Chromium platform of human intestine (Wang *et al.* 2020) and mouse lymph node (Gu *et al.* 2024) (Table 2). We randomly sampled from these values for 1000 cells of each cell cycle phase (G1, S, G2M) for each dataset. For each synthetic cell, we randomly selected one cell of the appropriate stage from the respective ground-truth cell cycle dataset and randomly sampled the specified number of reads from the read counts of the selected cell weighting each read equally, thus each gene is weighted by its total number of reads. This resulted in a similar number of genes detected per cell as in the original 10X datasets (Fig. 2, available as supplementary data at *Bioinformatics Advances* online). Undetected genes in the selected cell were not given a pseudocount, as original cells had to be down sampled to match the total UMIs per cell of the 10X datasets. Each cell cycle classification method was run on the resampled datasets and performance was evaluated as above. The MacDavid dataset was excluded since it did not measure the whole transcriptome, and the Sasagawa dataset was excluded due to the low number of G2M and S samples (<10 samples for each).

**Table 2:** Droplet-based benchmarking datasets.

Dataset	Tissue	Proliferative	Quiescent	Source
**Wang1**	colon	Transit amplifying	Paneth, Goblet	Data (Wang *et al.* 2020)
**Wang2**	ileum	Transit amplifying	Paneth, Goblet	Data (Wang *et al.* 2020)
Moreno	glioblast-oma	Malignant cells, radial glia	Oligodendrocyte, endothelial, plasma, astrocyte	Data (Ruiz Moreno *et al.* 2022)
**Street**	adipose	PRM1+ cells	Endothelial, smooth muscle, mature adipocytes	Data (Gupta *et al.* 2023)
**Gu**	lymph node	Germinal B-cells, plasmablast, prolif-DC, prolif-Treg	Memory B, plasma, naïve B, CD4 memory, endothelial, th17, naïve T	Data (Gu *et al.* 2024)
**Triana**	bone marrow	All progenitors, precursor B cells	Mature NK, T, plasma, naïve B, monocyte, memory B/T	Data (Triana *et al.* 2021)

#### 2.2.5 Droplet-based datasets

Annotated droplet-based single-cell datasets (Table 2) were downloaded by cellxgene (Megill *et al.* 2021). For CycleMix, datasets were normalized to the median total UMI counts across cells, the log2 transformed with a pseudocount of 1. For Seurat, we used the provided log2-normalized gene expression from cellxgene. Cyclone was run on raw counts. All methods were run on the complete dataset for the specified tissues. Cell types were classified as proliferative or quiescent or unknown based on known biology and confirmed by inspecting the expression of *TOP2A* and *HGMB2*. Cyclone was not run on the Moreno dataset as this dataset contained >100 000 cells, making it computationally prohibitive to run Cyclone on it.

Local purity of cell cycle phases was calculated to account for cell type such as malignant tumor cells, where it is expected that only a subset of cells would be actively cycling. *K*-nearest neighbors of each cell were calculated using Seurat with a *k* = 50. For each cell of the appropriate phase, we calculate what proportion of their neighbors were assigned to the same phase. As the datasets contained over 4000 cells, *k* = 50 represents a more local specific cell population that the broad cell type classifications.

#### 2.2.6 Assessing effect of normalization

To assess the effect of normalization on cell cycle classification, we normalized raw UMI counts using either log base 2 counts per million rescaling each cell to the median total UMIs per cell in that dataset, or using sctransform(v0.4.0) with default parameters (Hafemeister and Satija 2019).

#### 2.2.7 Assessing effect of sparsity

To assess the effect of sparsity on cell cycle classification, we randomly down sampled the Wang colon dataset using scuttle (v1.4.0). Data down sampled to 5% of the original total UMIs per cell were then smoothed using the KNN approach described above while varying the threshold for the proportion of nearest neighbors required to be classed as cycling to classify a given cell as cycling.

#### 2.2.8 Assessing effect of marker gene choice

Each of the six droplet-based datasets was classified using CycleMix with each of the five gene sets. Human and mouse genes were converted as appropriate using Ensembl biomart orthology relationships. Overlap was assessed as the intersection of each pair of gene sets divided by the maximum size of the two gene sets. Log fold enrichment for individual cell cycle phase pairs was calculated as the average across datasets of the proportion of cells assigned to a specific phase pair divided by the expected if cells were randomly classified then log transformed.

#### 2.2.9 Regressing cell cycle

All droplet-based datasets (Table 2) were subject to cell cycle regression using both Seurat and CycleMix. For Seurat, we used sctransform (v0.4.0) including the phase scores for S and G2M in the model as the creators recommend. For CycleMix, we specifically regressed differences between S and G2M cells while preserving differences between cycling and non-cycling cells (see above). Seurat requires datasets to be randomly down sampled to no more than 5000 cells for cell cycle regression, thus, we did the same when running the CycleMix regression to ensure consistency. For CycleMix, we down sampled to ensure equal presentation of each cell cycle phase. Fit coefficients were used to regress the cell cycle from all cells. We then assessed the purity of neighborhoods around G2M cells using the method described above. We assessed both purity of G2M cells alone to evaluate separation between S & G2M cells and purity of either G2M or S cells (i.e. all proliferating cells) to assess whether differences between cycling and non-cycling cells were preserved by each method.

In addition, we considered how well preserved the cell type distinctiveness was after cell cycle regression. To improve sensitivity, we considered only the proliferative cell types as non-cycling cells should not be affected by cell cycle regression. We considered both purity, as defined above, and the local inverse Simpson index (LISI) using the lisi(v1.0) package on the proliferating cell types to assess how well the distinctiveness of these cell types was preserved.

For each dataset, we compared the inferred frequency of cycling cells (S/G2M) from each method between proliferative and quiescent cell types using a two-sided proportion test. Cell types with fewer than 50 cells were excluded from analysis. Proliferation scores were calculated for each cell based on the highest phase score from either S or G2M and distributions of proliferative scores were compared using a KS test.

### 2.3 Quiescence

Marker genes of quiescence were obtained from (Cheung and Rando 2013), and simplified to up (weight = 1) and down (weight = −1) and added to the Tirosh dataset of cell cycle genes. CycleMix was used to classify each cell using this combined set of marker genes. Mouse orthologs of the Cheung genes were used for mouse datasets. The top quiescent cell types were identified as those with the highest proportion of quiescent cells, excluding all cell types with fewer than 50 cells.

Novel quiescence-associated genes were identified using a general linear model applied to log2 normalized expression values, including both sample ID and cell type ID as covariates. Consensus genes were defined as those significantly up or down regulated in quiescent cells in 4/6 datasets. Pathway enrichments were determined using Fisher’s exact test on GO biological processes, Reactome, MSigdb Hallmark, and Biocarta pathways.

### 2.4 iPSC-derived macrophages

Single-cell RNAseq data from iPSC-derived macrophages was obtained from the Gene Expression Omnibus (GSE250618). Data were filtered and normalized as previously described (Kent *et al.* 2024). Seurat was run using the developer’s provided cell cycle marker genes. CycleMix was run using the Tirosh cell cycle gene set augmented with the G0 marker genes from Cheung and Rando (2013). Fetal myeloid cell data were obtained from https://developmental.cellatlas.io/fetal-immune, log-normalized and cell cycle classification were performed using Seurat and CycleMix (Tirosh genes). Frequency of proliferating cells (G1S/S or G2M) for Kupffer cells and non-specific macrophages was calculated for each time point where data were available converted into days post-conception.

## 3 Results

**Figure 1:**
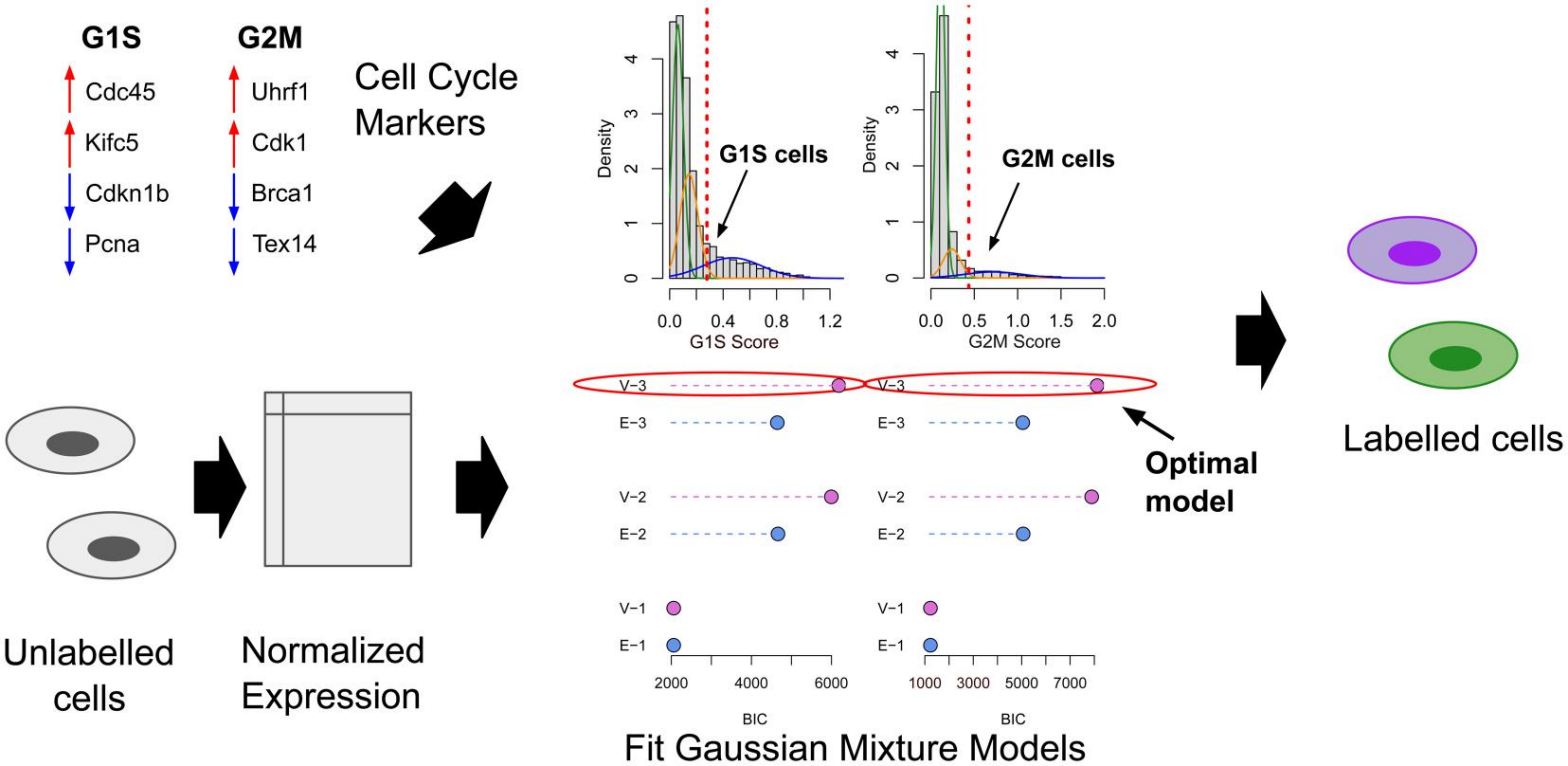
Diagram of the CycleMix approach. Log2-normalized expression is calculated for each cell, optionally any other normalization such as batch effect correction are applied. The weighted average of the normalized expression across up and down regulated markers of each cell cycle phase. Mixture models of 1, 2, or 3 Gaussian distributions are fit to the average scores across cells. The Bayesian information criterion (BIC) determines the optimal model to describe the data. Cells assigned to the Gaussian distribution, based on maximum posterior probability, with the highest mean for a phase are assigned to that phase.

CycleMix classifies cells into cell cycle phases using a variation on cell cycle scoring used by others (Macosko *et al.* 2015, Satija *et al.* 2015, Tirosh *et al.* 2016b). Raw unique molecular identifiers (UMIs) or read counts are library size normalized to the median library size across cells, then log transformed (Fig. 1). Phase scores are calculated using a weighted mean enabling both up and downregulated markers for each phase. Marker gene lists from multiple sources are available within CycleMix including those used by Seurat, Macosko *et al.* (2015), Tirosh *et al.* (2016b), and defined by Whitfield *et al.* (2002). As noted by others, we find the Tirosh dataset works well and use it by default in this article (Chervov and Zinovyev 2022). Whitfield contains signatures for more fine-scale dissection of the cell cycle, however these are often less cleanly separable in noisy single-cell data.

Scores of each phase are then fit to six different GMMs corresponding to mixtures of 1, 2, or 3 Gaussian distributions with either equal or variable variance. The Bayesian Information Criterion (BIC), which incorporates both the likelihood of the data given the model and the number of parameters used to fit the model to the data, is used to select the optimal Gaussian distribution model. Typically, the three Gaussian mixture variable variance model is chosen with distributions corresponding to cells in the specific phase with the highest average scores. Cells that are cycling but not in this specific phase are often found in the middle distribution and non-cycling cells typically have the lowest mean score (Fig. 1, available as supplementary data at *Bioinformatics Advances* online). Cells are individually assigned to the distribution with the highest posterior probability, and the cells assigned to the distribution with the highest mean are assigned to that phase. To resolve cells assigned to multiple phases in this initial step, phase scores are *Z*-transformed and cells are assigned to the phase with the highest *Z*-score. This *Z*-scoring approach accounts for the potential different number of genes or different capture rate of the genes for different phases.

Gaussian or negative binomial regression models can then be used to remove differences between any set of cell cycle phases the user would like, enabling removal of cell cycle phase differences while preserving differences between cycling and non-cycling cells.

### 3.1 Benchmarking on datasets with known cell cycle phase

**Figure 2:**
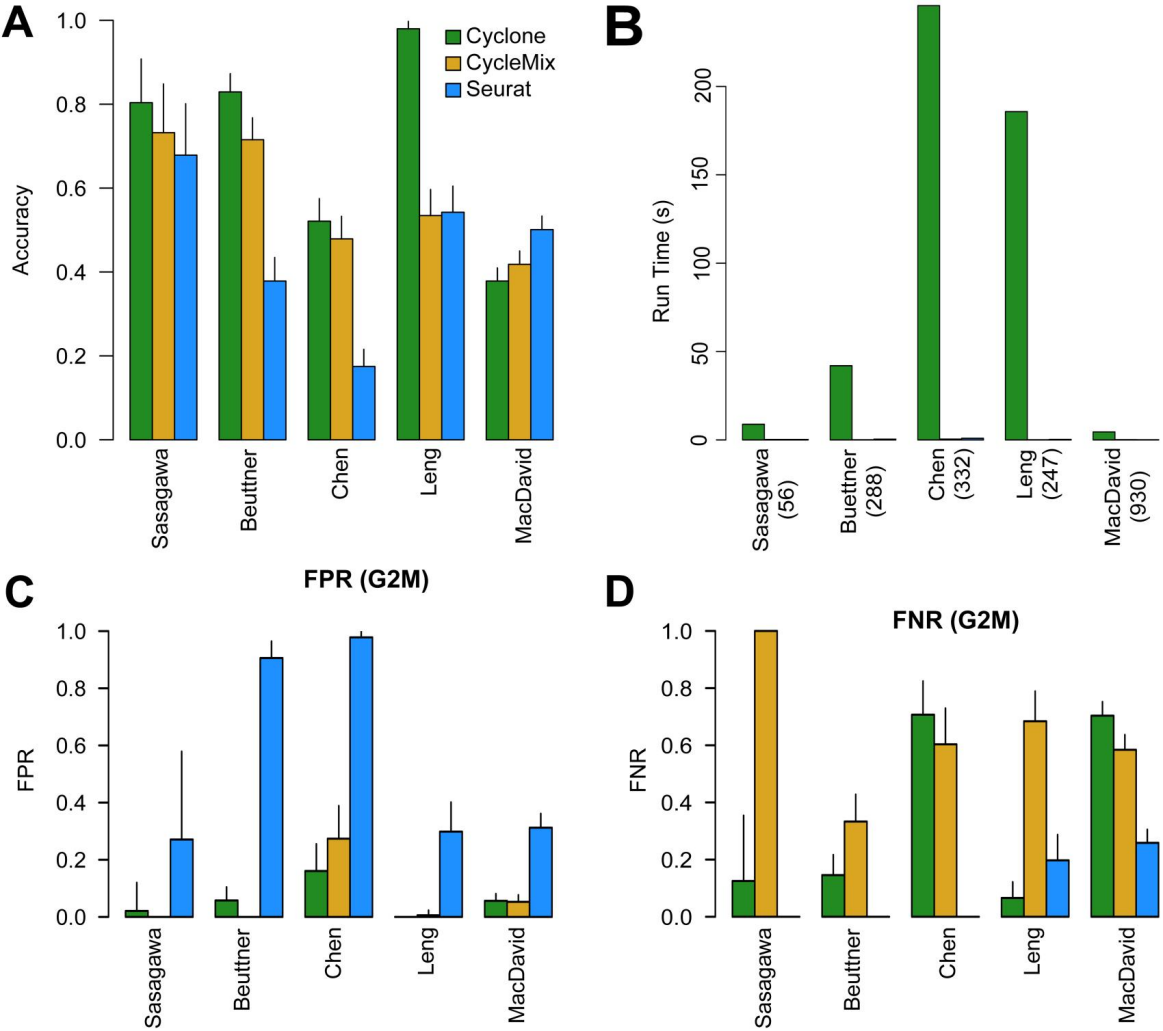
Benchmarking on five datasets with orthogonally determined cell cycle phase. (A) Overall accuracy of cell cycle stage predictions. (B) Runtime of each method on each datasets. (C) Relative number of G1 or S cells assigned to G2M. (D) Proportion of G2M cells assigned to G1 or S. Green = Cyclone, Gold = CycleMix, Blue = Seurat. Bars indicate 95% CI.

To validate the effectiveness of CycleMix compared to other cell cycle inference methods, we obtained five publicly available datasets where the cell cycle stage was assessed using an orthogonal method by synchronizing cells, flow sorting using cell shape features, or using fluorescent reporter genes (Table 1). Each dataset was classified into cell cycle phases using CycleMix, Seurat (Satija *et al.* 2015), and Cyclone (Scialdone *et al.* 2015). Overall, Cyclone had the highest accuracy in classifying single cells across all datasets (Fig. 2 and Fig. 2, available as supplementary data at *Bioinformatics Advances* online), with CycleMix a close second, whereas Seurat performed no better than random chance on both Buettner and Chen datasets. While Cyclone was most accurate as it was also much less scalable than CycleMix or Seurat (Fig. 2B) requiring 4 min to classify the 332 cells of the Chen dataset versus less than 10 s for CycleMix or Seurat.

Seurat consistently overestimated the proportion of mitotic cells (Fig. 2C), assigning 35%–98% of cells to the G2M stage across the datasets. In contrast, CycleMix was more conservative, and tended to underestimate the proportion of mitotic cells (Fig. 2D). Cyclone was inconsistent, overestimating the number of S-stage cells in the Leng and Sasagawa, but underestimating both S and G2M in Chen and MacDavid datasets.

#### 3.1.1 Downsampling datasets to mimic droplet-based technologies

**Figure 3:**
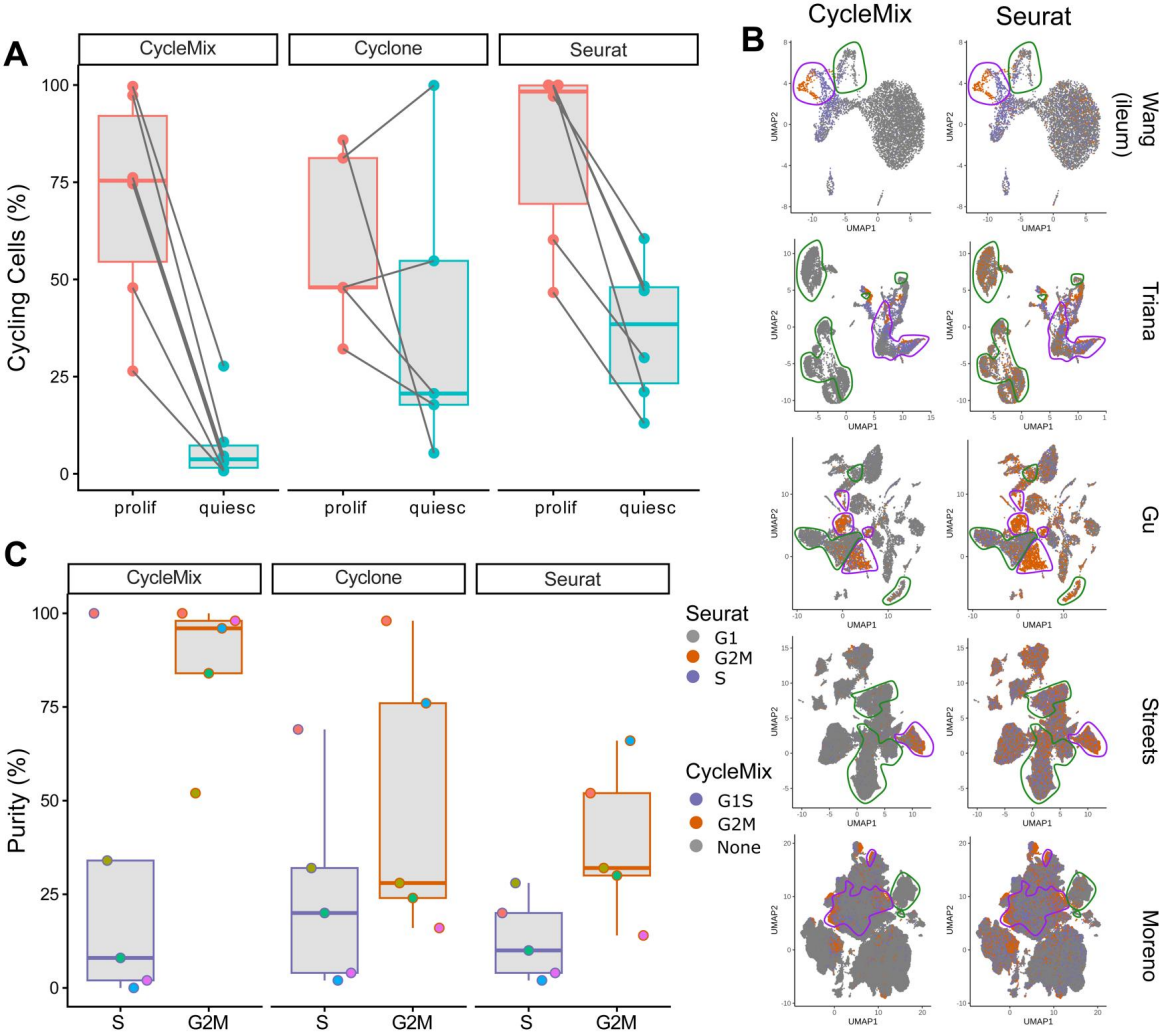
Assessing performance on droplet-based datasets with known proliferative and quiescent cell populations. (A) Proportion of cells classified as S or G2M by each method that were annotated as belonging to a proliferative or quiescent cell type. Lines connect matching points for each dataset. (B) UMAPs depicting cell cycle classification by CycleMix (left) and Seurat (right) for the different datasets. Purple and green outlines indicate the proliferative and quiescent populations, respectively. (C) Local purity of cell type classifications.

While the above datasets have reliable ground-truth cell cycle stage information available, the cells were captured and sequenced using plate-based (Sasagawa *et al.*, 2013, Chen *et al.*, 2018) and microfluidic chip based methods (Buettner *et al.* 2015, Leng *et al.* 2015) without the use of UMIs. As a consequence, these datasets are much smaller (56–930 cells), have different noise profiles and sensitivities than current state-of-the-art droplet-based methods. To assess the performance of the methods on data more similar to modern droplet-based methods, we repeatedly downsampled the transcripts and upsampled the number of cells for three of these datasets (Fig. 3, available as supplementary data at *Bioinformatics Advances* online). Results were similar to those observed for the original datasets with CycleMix and Cyclone performing well on the Buettner datasets, none performing well on the Chen dataset and Cyclone outperforming the others on the Leng dataset. While this downsampling accounted for sensitivity and cell-number differences it did not account for different noise profiles between UMI-based platforms which follow a negative binomial distribution, and plate-based methods which follow a zero-inflated negative binomial distribution (Pierson and Yau 2015, Svensson 2020, Jiang *et al.* 2022).

### 3.2 Benchmarking on droplet-based scRNAseq datasets

To benchmark the cell cycle classification methods on state-of-the-art scRNAseq datasets, we collected six publicly available datasets containing some cell types known to be highly proliferative and some cell types known to be quiescent (Table 2). We ran each cell cycle classification method on all cells in each dataset and assessed the proportion of cells assigned to S/G2M phases among the mature non-proliferative cell types and the expected highly proliferative cell types (Fig. 3A). We found that both CycleMix and Seurat but not Cyclone consistently assigned a higher proportion of cells to S/G2M for the expected proliferative cell types than the quiescent cell types. This suggests that Cyclone may suffer from being trained on full-length Smartseq2 scRNAseq and does not perform well on 3′ or 5′ UMI-based scRNAseq datasets. However, only CycleMix accurately assigned more than >90% of quiescent cells to a non-cycling phase, whereas Seurat assigned 25%–50% of quiescent cells to S/G2M.

**Figure 4:**
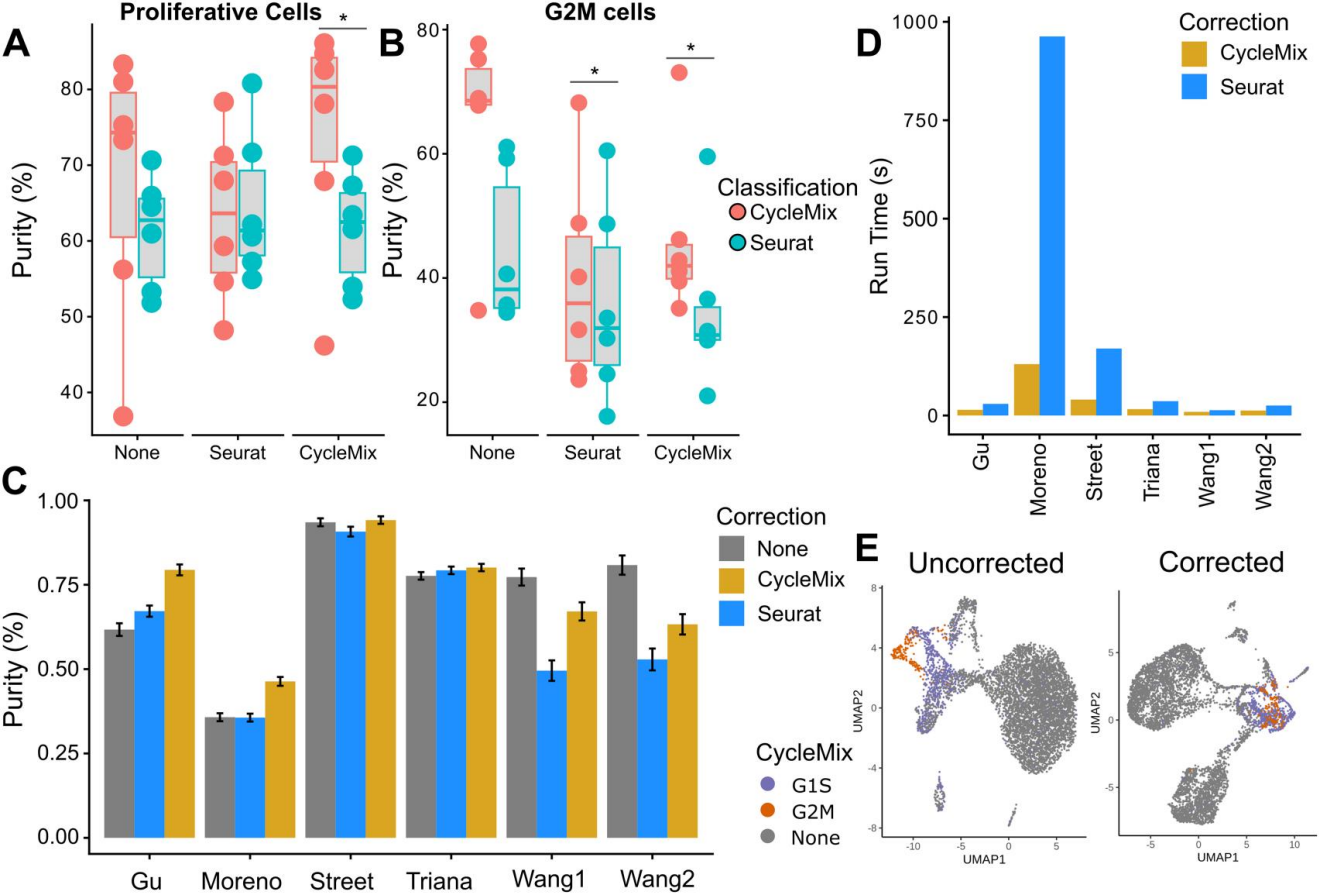
Partial cell cycle regression preserved distinctiveness of proliferative cell-types. (A) CycleMix regression preserves separation of G2M/S cells from non-proliferating cells. Each dot represents one dataset, colors indicate the origin of the cell cycle labels used to determine G2M/S cells, None = uncorrected, Seurat = Seurat CC regression. CycleMix = partial CC regression included in CycleMix. Stars indicate significant differences compared to no-regression (*P* < .05, paired *t*-test) (B) As in A but considering only purity of G2M cells within themselves. (C) CycleMix regression preserves or enhances separation of proliferative cell types. Average purity of each proliferative cell type. Error bars indicate 95% confidence intervals. (D) Runtime for CycleMix and Seurat regression on each dataset. (E) UMAPs before and after CycleMix partial regression for the Wang ileum dataset. Equivalent figures for the other datasets are in Fig. 8, available as supplementary data at *Bioinformatics Advances* online.

**Figure 5:**
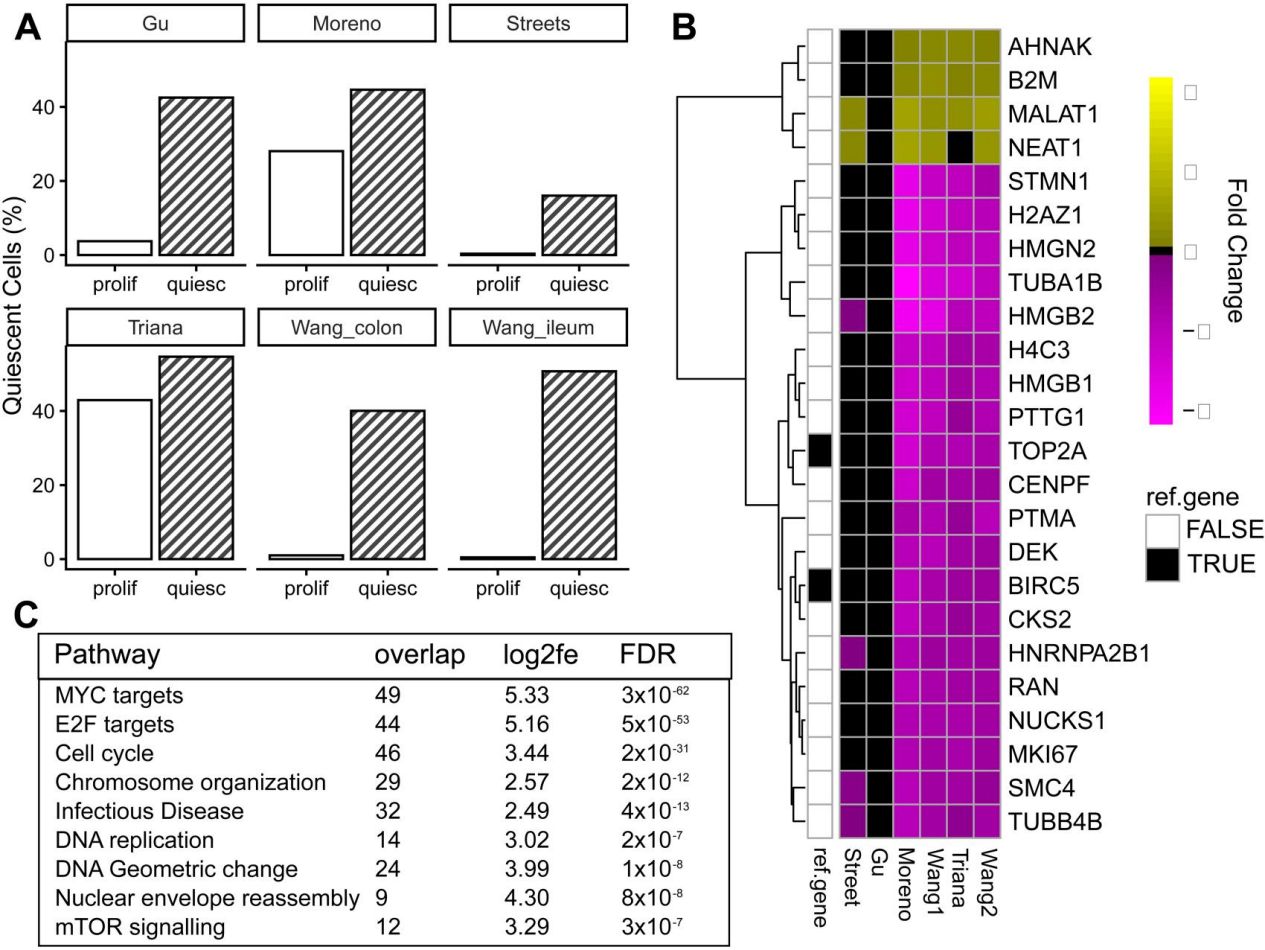
CycleMix can identify G0 cells. (A) Proportion of cells labelled as quiescent among cell types that should be proliferative or quiescent in each dataset. Quiescent genes were obtained from Cheung and Rando (2013). (B) Top most consistently differentially expressed genes between quiescent and proliferative cells across datasets. ref.gene indicates whether it was among the genes identified in Cheung and Rando. (C) Top significantly enriched pathways among genes downregulated in quiescent cells. Log2fe = log 2 fold enrichment.

The GMM used by CycleMix was a good fit to these datasets (Fig. 4, available as supplementary data at *Bioinformatics Advances* online). We confirmed the good fit by randomly sampling from the fitted distribution and calculating the Kolmogorov–Smirnov statistic (*D*), all fits achieved an average *D* < 0.2 over 100 random samples, and most achieved an average *D* ∼ 0.05. In addition, we confirmed that our choice of data normalization had little effect on the cell classifications (Fig. 5A, available as supplementary data at *Bioinformatics Advances* online). Cell cycle classifications were highly robust, with performance stable even when datasets were downsampled to 20% of the original total UMIs (Fig. 5C, available as supplementary data at *Bioinformatics Advances* online).

**Figure 6:**
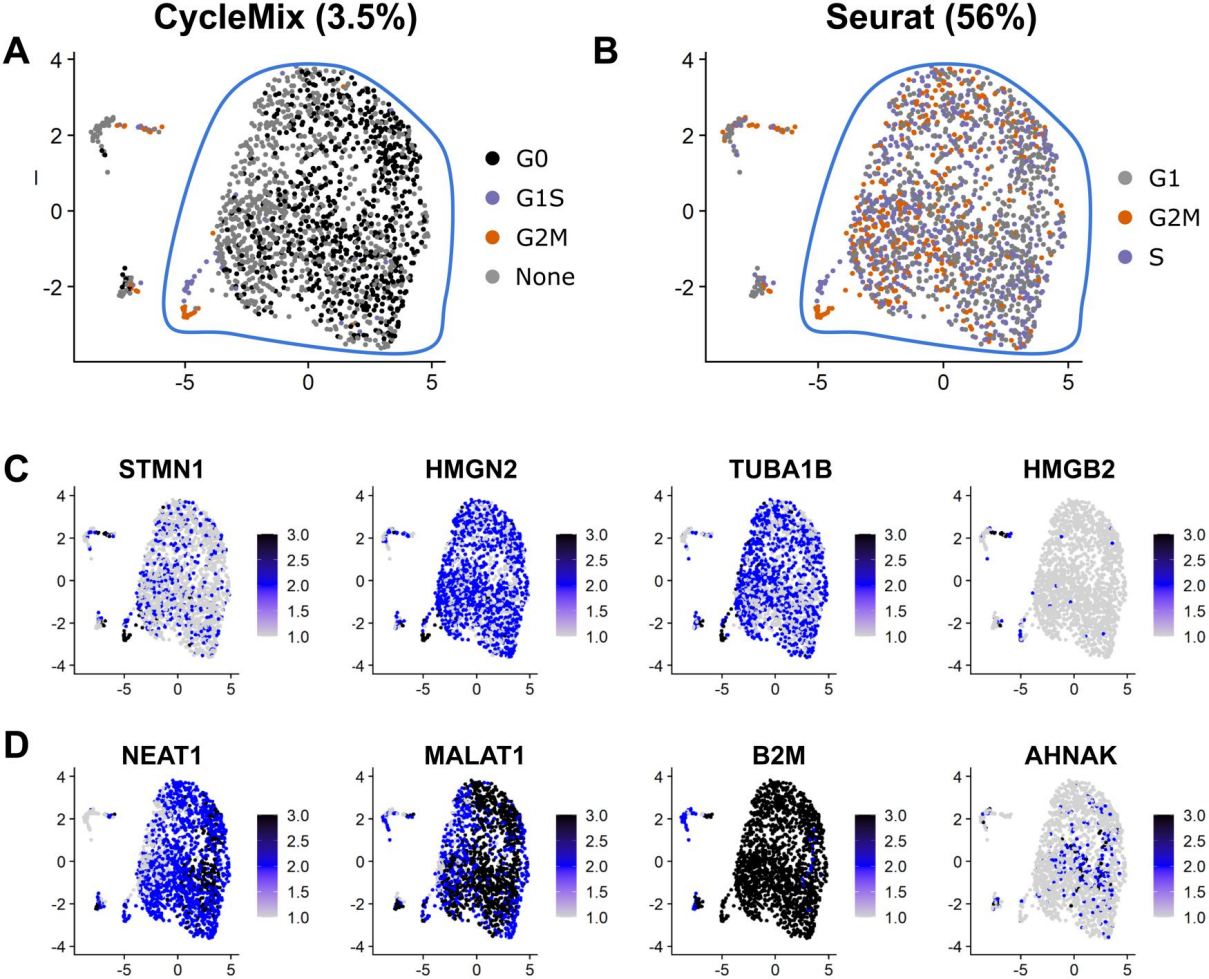
CycleMix identified subpopulation of proliferating iPSC-derived Kupffer cells. (A) Cell cycle classification of iPSC-derived Kupffer cells (blue circle) using CycleMix. (B) As in (A) but with Seurat classifications. (C) Expression of the top four downregulated quiescence genes (D) Expression of the top four up regulated quiescence genes.

The choice of cell cycle marker genes can also affect cell cycle classifications (Fig. 6, available as supplementary data at *Bioinformatics Advances* online). Of the five sets provided within CycleMix from the literature, Seurat and Tirosh were identical with only two phases defined, and Cyclone defining three phases, whereas the Whitfield, and its subsetted version from Macosko defined five and six phases, respectively. However, similar proportions of cells were classified as proliferative when “G1” and “MG1” phases were considered as non-proliferative states for five of our six datasets with only large discrepancy in the Streets-Adipose dataset (Fig. 6A, available as supplementary data at *Bioinformatics Advances* online). Cell cycle phase assignments were also consistent between these marker gene sets with Cyclone-derived gene sets having the most discrepancies compared to the other gene sets (Fig. 6C, available as supplementary data at *Bioinformatics Advances* online). Despite the Whitfield/Macosko geneset having little overlap with the Seurat/Tirosh geneset both arrived at similar classifications.

Since the cell cycle involves consistent changes in protein expression to perform DNA replication and then mitotic division, we reasoned that cells of the same cell type within the same stage of the cell cycle should cluster together in gene-expression space. Thus, we expect the local neighborhood around G2M cells to predominantly contain other G2M cells and likewise for cells currently in S-phase. Thus, we examined whether the local neighborhoods of each individual S and G2M cell contained other cells of the same phase (Fig. 3B and C). To avoid confounders such as batch effects or cell type differences causing S and G2M cells to be split apart, we only considered the 50 nearest neighbors of each cell.

For all methods, we observe that G2M cells were significantly more likely to cluster together than S-phase cells (*P* < 10–10, Wilcox-rank-sum test). This was most pronounced using the classifications obtained from CycleMix, where on average >50% of neighboring G2M cells were other G2M cells across all datasets, in contrast to ∼37% for Cyclone and ∼25% for Seurat. These results were consistent with Seurat exhibiting much higher false-positive rates for S and G2M cell type assignments, which would be randomly scattered across gene-expression space thus having few neighbors in the same phase.

#### 3.2.1 Smoothing to improve sensitivity

To improve the sensitivity and specificity of CycleMix, we applied *k*-nearest-neighbor smoothing (see Section 2). Using the standard 20 principal components and *k* = 20 used as defaults in most scRNAseq analysis pipelines. To enhance the sensitivity of CycleMix we required only 25% of neighbors being proliferative (G1S or G2M) to assign a cell as proliferative. This improved the sensitivity and specificity on the quiescent and proliferative cell types for five of the six datasets (Fig. 5B, available as supplementary data at *Bioinformatics Advances* online). This smoothing was capable of recovering performance even for extremely sparse datasets obtained from downsampling to only 5% of the original dataset (Fig. 5D, available as supplementary data at *Bioinformatics Advances* online).

Smoothing by assigning a whole cell type to a single cell cycle phase further improved performance using our metrics (Fig. 5B, available as supplementary data at *Bioinformatics Advances* online). However, such an approach reduces the ability to detect different phases of the cell cycle and relies on having a dataset where entire cell types are expected to be cycling which is rarely the case for scRNAseq datasets. Thus, we do not recommend this approach when the ground truth is not known.

#### 3.2.2 Cell cycle regression

The best approach to account for the cell cycle in scRNAseq data remains uncertain (Kiselev *et al.* 2019, Heumos *et al.* 2023). A common approach is to generate a quantitative score representing the similarity of the cell to each cell cycle stage then use regression models to subtract the predicted gene expression effects of those scores [e.g. Seurat (Satija *et al.* 2015)]. Alternatively, discrete classifications can be used for this regression. Other approaches have included factor decomposition followed by removal of cell cycle related factors (Buettner *et al.* 2015).

A key challenge for these methods is when the cell cycle is confounded with biological variation of interest. For instance, in samples containing a highly proliferative subpopulation of stem cells or reactive immune cells, regression models may not distinguish problematic cell cycle variability from the biologically relevant cell type variability. Within our CycleMix package, we include a partial regression model based on discrete classifications which only removes differences between S and G2M cell cycle phases while preserving differences between cycling and non-cycling cells (see Section 2).

To evaluate the performance of our regression approach in contrast to the phase-score regression method used by Seurat, we performed both types of regression on each of our six datasets (Fig. 4). Notably, the Wang colon, Wang ileum, and Adipose datasets have a single highly proliferative cell type thus the cell cycle phase score and phase classifications are highly confounded with cell type in these datasets.

We used the same local-clustering of different phases used above to assess whether G2M cells were intermingling with cells of other phases, an indicator of successful removal of cell cycle phase differences (Fig. 4B). Whereas, only CycleMix preserves the local-clustering of proliferating cells as separate from non-proliferating cells (Fig. 4A). The local inverse Simpson Index (LISI) similarly shows CycleMix preserves or enhances the separation (lower LISI) of proliferative cell types compared to Seurat in five of the six datasets (Fig. 7, available as supplementary data at *Bioinformatics Advances* online).

Comparing our discrete partial regression to the Seurat score-based full regression, we find CycleMix is more effective at intermingling G2M and S cells while preserving the integrity of highly proliferative cell types (Fig. 4). Both regression methods significantly increased the mixing of cells in different stages of the cell cycle (Seurat: *P* = 0.02, CycleMix: *P* = 0.01, Fig. 4B). Whereas only CycleMix preserved, and sometimes enhanced, the consistency of proliferative populations (Fig. 4A and C).

Datasets with only one proliferative cell type are most at risk of removal of cell type characteristic differences being removed during cell cycle regression due to confounding of cell cycle and cell type. Thus, we examined the effect of CycleMix partial regression and Seurat full regression on our three datasets with only one proliferative cell type (Fig. 8–11, available as supplementary data at *Bioinformatics Advances* online). Consistent with Fig. 4, we note that the proliferating cell types remain more distinct within fewer clusters following CycleMix partial regression compared to after Seurat full regression.

### 3.3 Identifying quiescent cells

CycleMix can be used to assign cells to any category defined by a set of up and/or down regulated genes. Using a recent publication that identified genes consistently associated with quiescence in three different stem cell populations (Cheung and Rando 2013), we used CycleMix to identify quiescent (G0) cells in each of our six droplet-based datasets (Fig. 5A). Cell types expected to be quiescent were enriched in these cells versus proliferative cell types as expected (Fig. 5A). The Triana and Morano datasets had a substantial number of cells within the proliferative cell types that were assigned to G0 which is a result of the classification of cell types that are known to be only partially proliferative as entirely proliferative for these datasets. For Morano, we assigned all malignant cells as proliferative as we expect tumor cells to be more proliferative than mature neurons and glia. However, it is well established that glioblastoma tumors contain populations of quiescent cells (Xie *et al.* 2022, Castillo *et al.* 2023), which may explain the quiescences we see in this dataset. The Triana dataset contains bone marrow cells, of which we classified all stem and progenitor populations as proliferative, however, it is known that some of these cells transition between active and quiescent states (Takayama *et al.* 2021, Lynch *et al.* 2024), thus we would expect some of them to be quiescent.

The G0 markers we used were defined based on stem cells thus may not be ideal for assessing quiescence in differentiated cells. We used MAST (Finak *et al.* 2015) to identify genes associated with quiescence after controlling for cell type and sample-specific effects in all our datasets. Only *AHNAK*, *B2M*, *MALAT1*, and *NEAT1* were consistently upregulated in quiescent cells (Fig. 5B). *MALAT1* and *NEAT1* are nucleus-specific transcripts which are associated with cell-integrity in scRNAseq experiments thus are unlikely to specifically identify G0 cells (Clarke and Bader 2024). In contrast, 152 genes were consistently downregulated in quiescent cells (Table 1, available as supplementary data at *Bioinformatics Advances* online), as expected 32% of these genes are known targets of Myc, a well known oncogene and pro-proliferation transcription factor (Dang 1999, Dhanasekaran *et al.* 2022, Matthews *et al.* 2022), and 29% were targets of the proliferation-associated E2F transcription factor (Ren *et al.* 2002) (Fig. 5C) suggesting that in differentiated cells, quiescence is mainly determined by down regulation of proliferation pathways.

### 3.4 Proliferation of iPSC-derived Kupffer cells

Kupffer cells are unique liver-resident macrophages that phagocytose worn out and damaged red blood cells in the liver. Kupffer cells are embryonically derived and locally self-renewed to maintain their population in the liver independent of the bone marrow (Gomez Perdiguero *et al.* 2015, Mass *et al.* 2023). Recently, we generated scRNAseq data of iPSC-derived Kupffer cells that were transplanted into a humanized mouse (Kent *et al.* 2024), these cells expressed gene signatures consistent with fully mature liver Kupffer cells and could phagocytose red blood cells, thus are expected to not be cycling. As these cells had recently colonized the host liver, some may not be fully differentiated and still be proliferating. Using CycleMix, we identified a distinct subpopulation of actively proliferating cells accounting for 3.5% of the Kupffer cell population, whereas Seurat classified 56% of the Kupffer cells as actively proliferating (Fig. 6A and B). Expression of the top positive and negative markers of quiescence identified above confirmed most cells were quiescent and only a small population was proliferating (Fig. 6C and D and Fig. 12, available as supplementary data at *Bioinformatics Advances* online). When compared to normal human development (Suo *et al.* 2022), iPSC-derived Kupffer cells were significantly less proliferative than normal human fetal Kupffer cells when using CycleMix (proportion test, *P* < .05), whereas using Seurat would lead to the opposite conclusion (Fig. 13, available as supplementary data at *Bioinformatics Advances* online).

## 4 Discussion

We present CycleMix, a novel scalable cell cycle classification algorithm based on GMMs. Briefly, this approach uses a weighted average log-normalized expression to combine positive and negative gene markers to generate stage-specific scores which are binarized into discrete classifications by fitting a mixture of Gaussian distributions and using the BIC to select the optimal model (Fig. 1). By dynamically fitting both cycling and non-cycling cells, CycleMix was able to more accurately distinguish non-cycling cells than the current state-of-the-art approaches, while maintaining a high degree of scalability. We demonstrated CycleMix’s performance on both gold- and silver-standard datasets from a range of different single-cell RNAseq technologies.

We observed that Cyclone outperformed CycleMix on most gold-standard benchmarking datasets (Fig. 2), but performed poorly on silver-standard datasets (Fig. 4). This was due to all current gold-standard datasets using full-length transcript sequencing protocols. Full-length scRNAseq has different mean expression levels, due to differences in gene-length (Phipson *et al.* 2017), and follow a zero-inflated negative binomial, in contrast to modern droplet-based protocols which have no gene-length bias and do not exhibit zero inflation (Korsunsky *et al.* 2019, Svensson 2020, Jiang *et al.* 2022). Cyclone was trained exclusively on full-length Smartseq2 scRNAseq datasets, to identify gene pairs where the relative expression between the genes changes between cell cycle phases (Scialdone *et al.* 2015). The relative expression of these gene pairs is expected to be substantially altered when measured with droplet-based scRNAseq protocols leading to the observed poor performance.

Current state-of-the-art scRNAseq uses almost exclusively 3′ or 5′ tag-based scRNAseq datasets, when we tested all three classification methods on such data CycleMix clearly outperformed Seurat and Cyclone. In contrast, the classification method in the popular Seurat package demonstrated very high false-positive rates. Seurat on average classified 40% of quiescent cells being classified as cycling, in contrast to <10% for CycleMix (Fig. 3). This was consistent with results on the full-length gold-standard datasets where Seurat classified an average of 55% non-G2M cells as G2M, versus 6% for CycleMix and 5% for Cyclone (Fig. 2). Seurat uses a flat threshold of a mean G2M or S score of 1 to classify a cell as cycling, in contrast, CycleMix adaptively determines an appropriate threshold for each dataset, which may explain Seurat’s high false-positive rate.

It has been noted that there are two different forms of the cell cycle in mammalian cells: a fast version with minimal G1 phase used by ESCs and cancer, and one with a significant G1 used by normal adult tissues (Becker *et al.* 2006, Coronado *et al.* 2013, Chervov and Zinovyev 2022). This fast cell cycle may directly contribute to maintaining pluripotency by blocking the transcription of long genes associated with differentiation (Abou Chakra *et al.* 2021). Three of the five gold-standard datasets originate from ESCs or induced pluripotent cells, and a fourth used cancer-derived cell-lines. In contrast, our silver-standard datasets were predominantly healthy adult tissues with only a single cancer dataset. The gene list we used for cell cycle classification was derived from Tirosh *et al.* (2016a) which examined metastatic cancer, thus likely represent the fast version of the cell cycle which may explain why the G2M classifications were more consistent and concentrated within the datasets than the G1S phase cells (Fig. 3C). The choice of cell cycle markers has a significant effect on cell cycle classification (Fig. 6, available as supplementary data at *Bioinformatics Advances* online), thus classification accuracy for CycleMix could be further improved by identifying cell cycle phase markers derived from cycling normal adult cells.

Using a recently published set of quiescence markers (Cheung and Rando 2013), CycleMix could classify cells across all tissues as G0 versus G1S or G2M (Fig. 3), however, these classifications were primarily driven by down regulation of various proliferation markers rather than any consistent positive markers of G0 across tissues. The only transcripts consistently associated with G0 were two nuclear-associated long-noncoding RNAs and *AHNAK* and *B2M*. *AHNAK* has been previously associated with anti-proliferative effects in cancer (Lee *et al.* 2014, Chen *et al.* 2017), but it is unclear if it has a direct interaction with cellular proliferation machinery (Davis *et al.* 2014). *B2M* is a member of the major histocompatibility complex (MHC-I), which may have indirect effects on suppressing proliferation (Smith *et al.* 2015). Thus, it is unclear whether there are any consistently upregulated genes associated with quiescence across all tissues and cell types.

While we have shown that CycleMix significantly outperforms existing methods of cell cycle assignment, its reliance on fitting GMMs makes it a poor choice for datasets with small numbers of cells (<1000). It also relies on the cell cycle phase score following a Gaussian distribution, thus we have included in the package a bootstrapping approach to assess the quality of the fit of the Gaussian distribution in addition to the BIC. While most normalization methods will result in Gaussian distributed data, users should be cautious when using CycleMix on imputed or batch effect corrected values as some of these methods are known to distort expression value distributions (Andrews and Hemberg 2018, Hou *et al.* 2020, Antonsson and Melsted 2024).

Thus, we have created a more accurate and more flexible single cell cycle classification approach compared to the current state-of-the-art. We demonstrated the importance of accurate cell cycle classification when considering determining the maturity of iPSC-derived cells. Other instances where accurate absolute quantification of proliferation is important is in cancer biology, due to known correlations between high tumor proliferation and poor patient prognosis (Li *et al.* 2021, Nagy *et al.* 2021), and potential importance for inflammation-associated disease as proliferation of lymphocytes and subsequent suppression is a key step in the immune response (Wang *et al.* 2024). We have shown that only CycleMix can accurately estimate the frequency of proliferating cells within scRNAseq datasets.

## Supplementary Material

vbag179_Supplementary_Data

## Data Availability

The list of accession numbers for benchmarking datasets is available in Table 1, real 10X datasets used for benchmarking (Table 2) are available in cellxgene at the provided links. iPSC-derived macrophage data are available in Gene Expression Omnibus (GSE250618). Fetal macrophage data are available at https://developmental.cellatlas.io/fetal-immune.
