# Supplementary material for: CycleMix: Gaussian mixture modeling of the cell cycle
Source: Bioinform Adv. 2026 Jun 22;6(1):vbag179. doi: 10.1093/bioadv/vbag179 (PMC13479747; doi:10.1093/bioadv/vbag179)
Supplement: Supplementary file 1 [file vbag179_supplementary_data.zip › CycleMix_2026_Supplementary_Revision.pdf]

Supplementary Figures

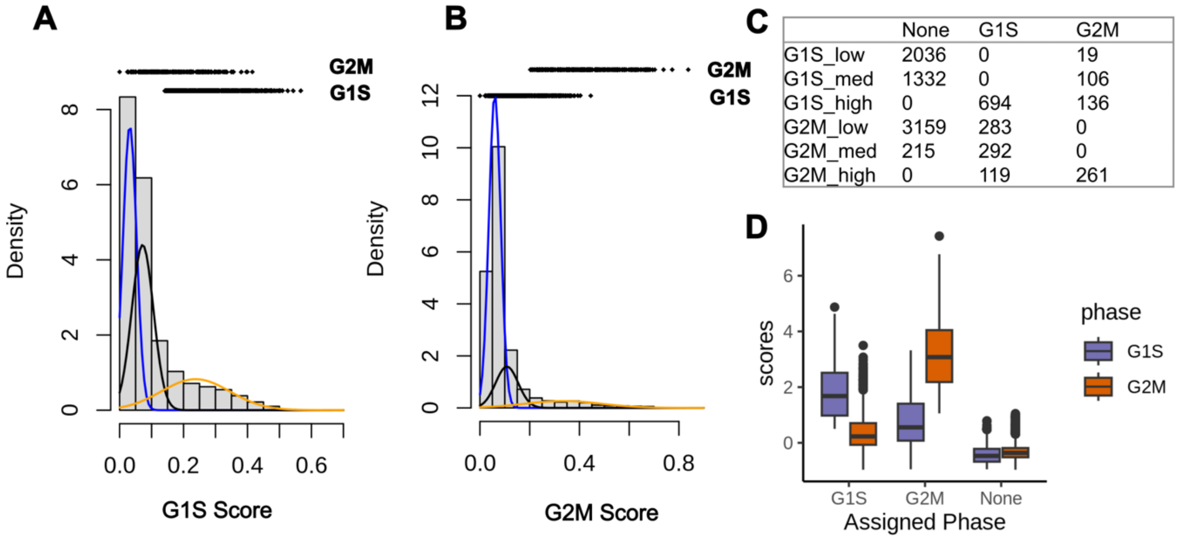

	None	G1S	G2M
G1S_low	2036	0	19
G1S_med	1332	0	106
G1S_high	0	694	136
G2M_low	3159	283	0
G2M_med	215	292	0
G2M_high	0	119	261

Figure S1: Colonic cells from the Wang dataset were classified into cell-cycle phases by CycleMix (A) Optimal Gaussian mixture model fit to the G1S scores. (B) Optimal Gaussian mixture model fit to the G2M scores. Dots indicate the final phase assignment of each cell. (C) Table showing the number of cells assigned to each of the three Gaussian distributions in A and B, that were assigned to each final phase. (D) Boxplot of the G2M and G1S scores for cells assigned to each phase.

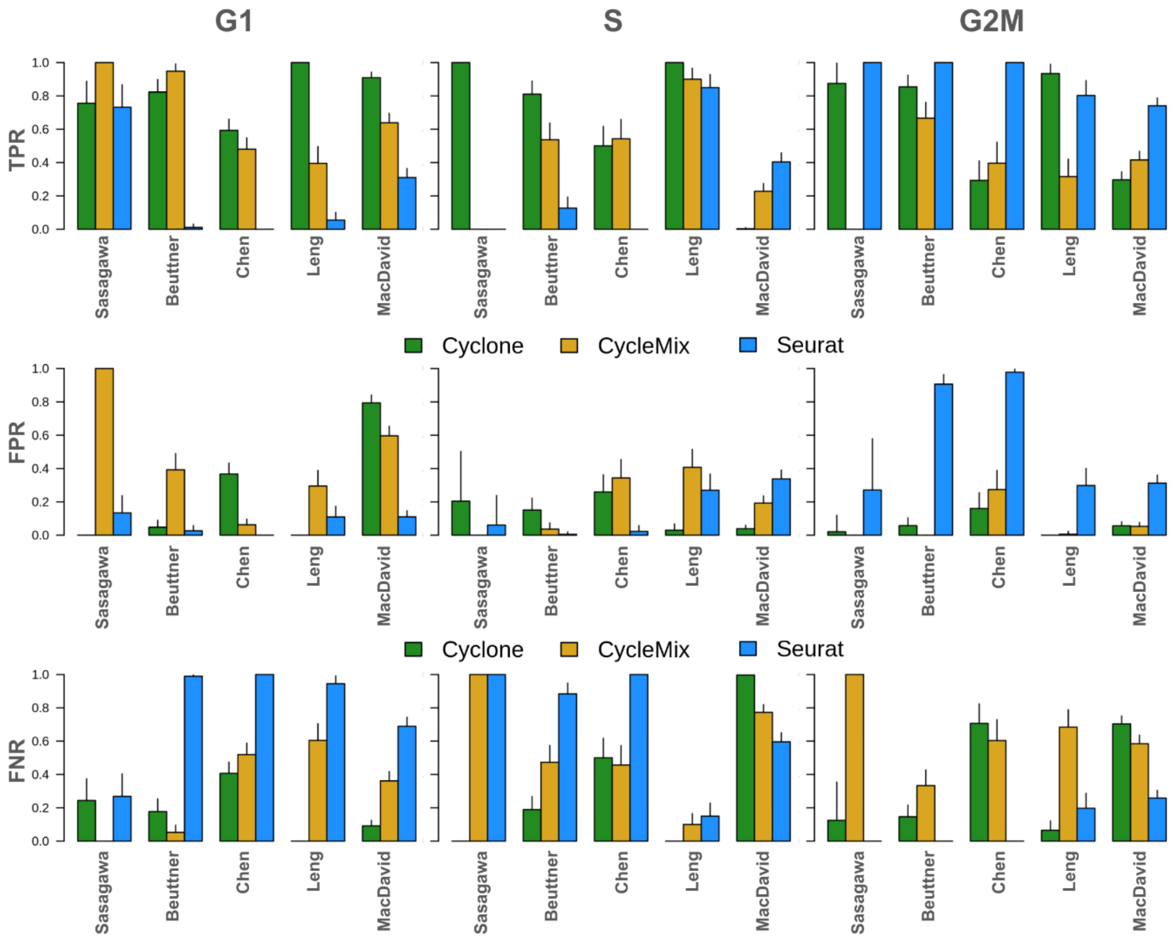

Figure S2: Accuracy of cell classifications on gold-standard ground truth datasets. (top) true positive rate (middle) false positive rate (bottom) false negative rate of each cell-cycle stage G1 (left), S (middle), and G2M (right)

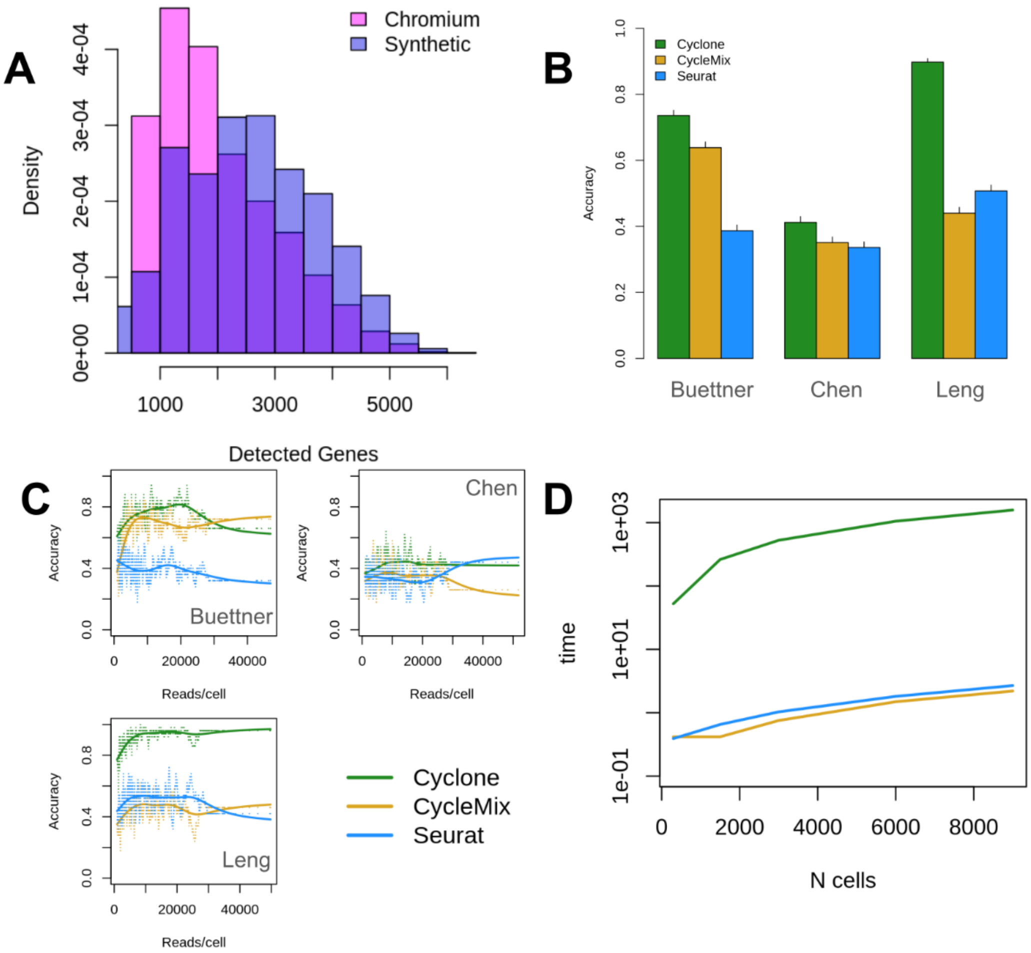

Figure S3: Benchmarking on resampled datasets to mimic droplet-based methods. (A) Downsampling of real datasets resulted in similar total detected genes per cell as 10X Chromium. (B) Cyclone generally outperformed other methods on all three downsampled datasets. Bars indicate 95% CI. (C) Relationship between classification accuracy and total reads / cell. Each point is the average accuracy over 50 cells, and lines are spline fits to the trend across cells. (D) Cyclone was >500x slower than CycleMix or Seurat, requiring 30 minutes per 10,000 cells vs < 5 seconds per 10,000 cells for Seurat or CycleMix.

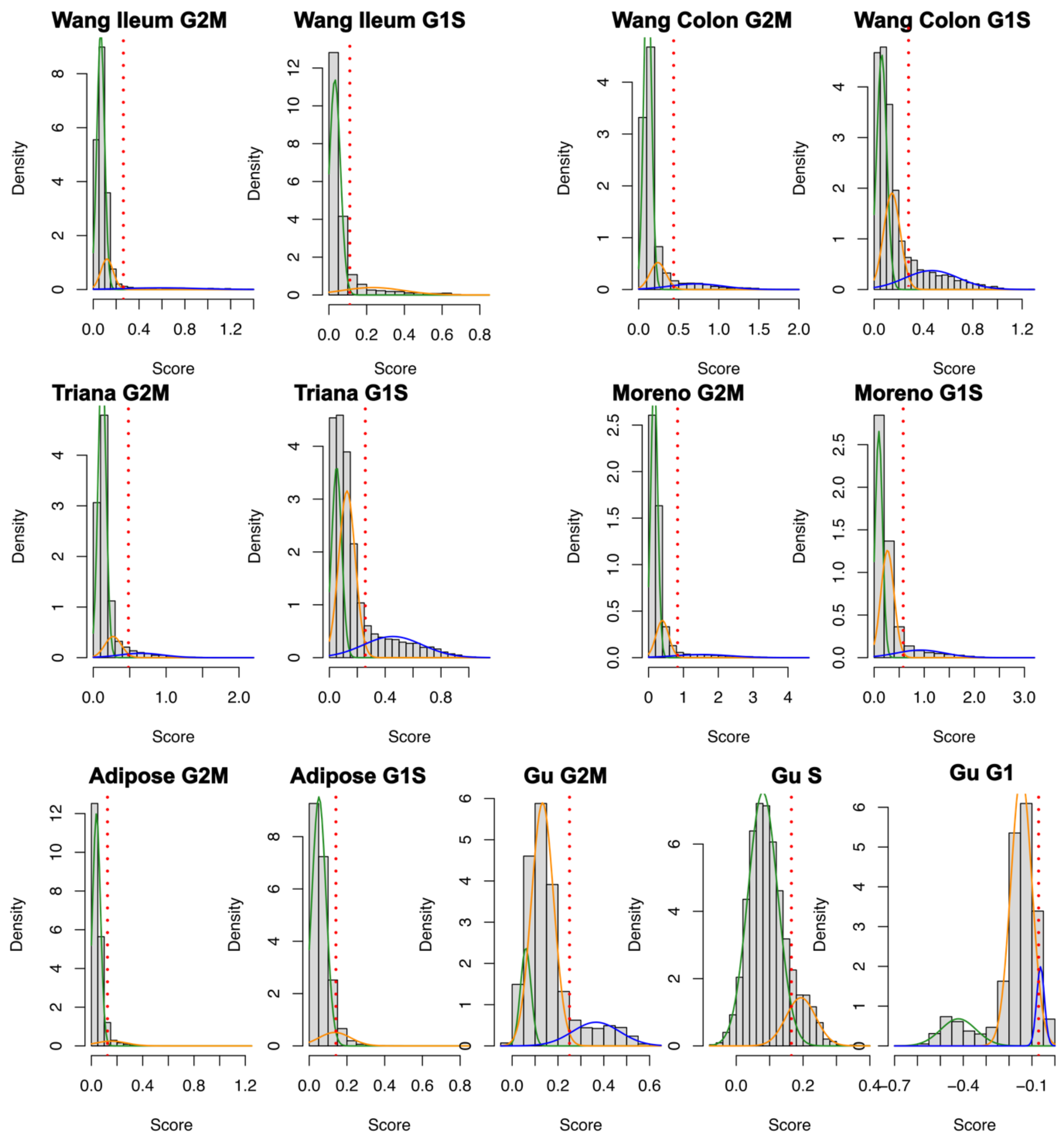

Figure S4: Gaussian mixture model fits for each phase for each UMI-based dataset. Gu was a mouse dataset so cell-cycle markers were obtained from cyclone for G1, S, and G2M. For all others, the Tirosh geneset was used to identify G1S and G2M cells.

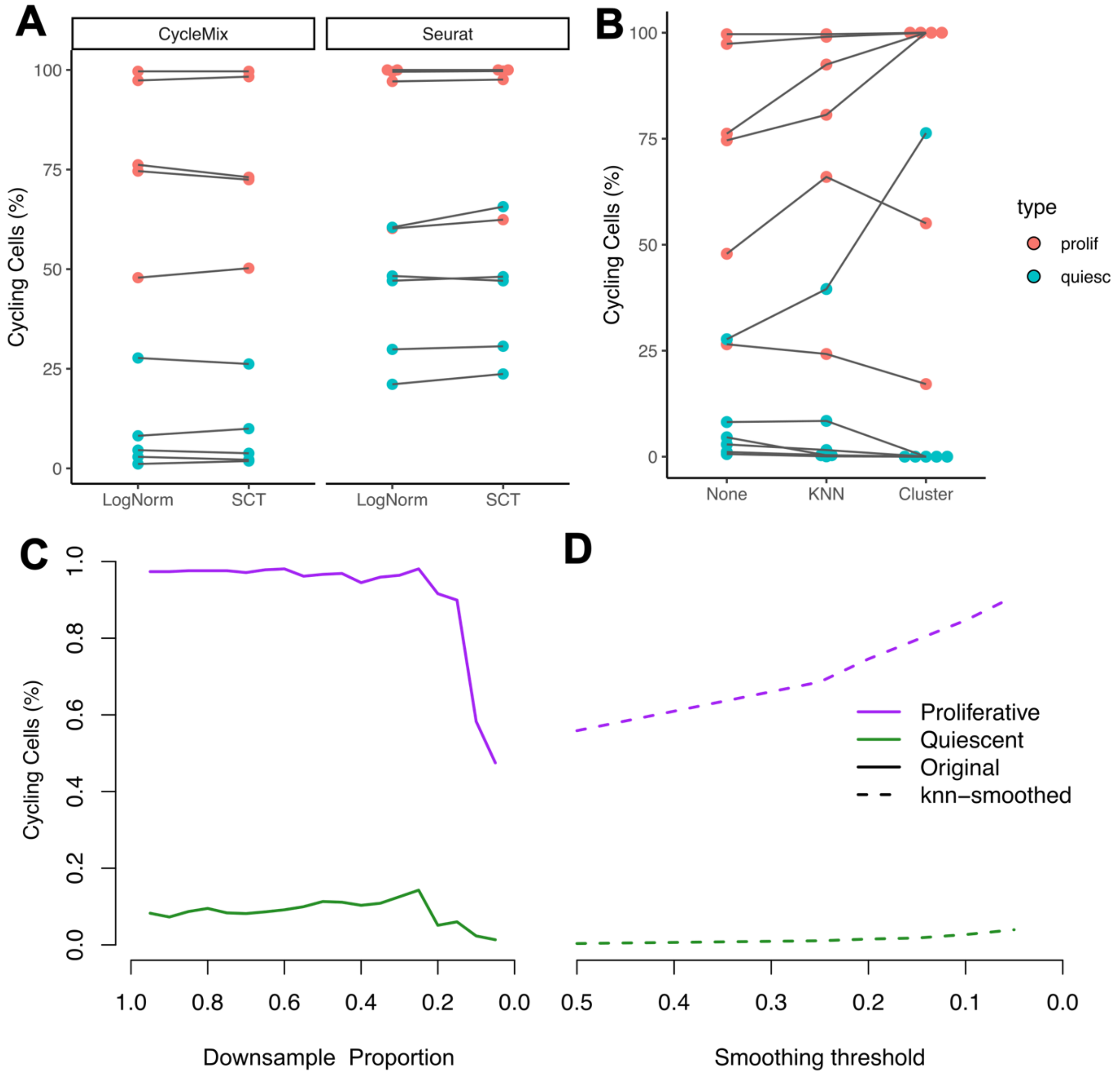

Figure S5: Testing the limits of CycleMix. (A) Normalization methods have little effect on performance. SCT = sctransform, LogNorm = log2 counts per million with pseudocount of 1. (B) Smoothing CycleMix results with k-nearest neighbours (KNN) or by cell-type (Cluster). (C) CycleMix phase classifications of proliferative and quiescent cell-types after down sampling to retain only a fraction of the original UMIs. (D) CycleMix classifications after using KNN smoothing on the 5% down sampled data, using different thresholds for the proportion of neighbours that must be cycling for a cell to be assigned as cycling.

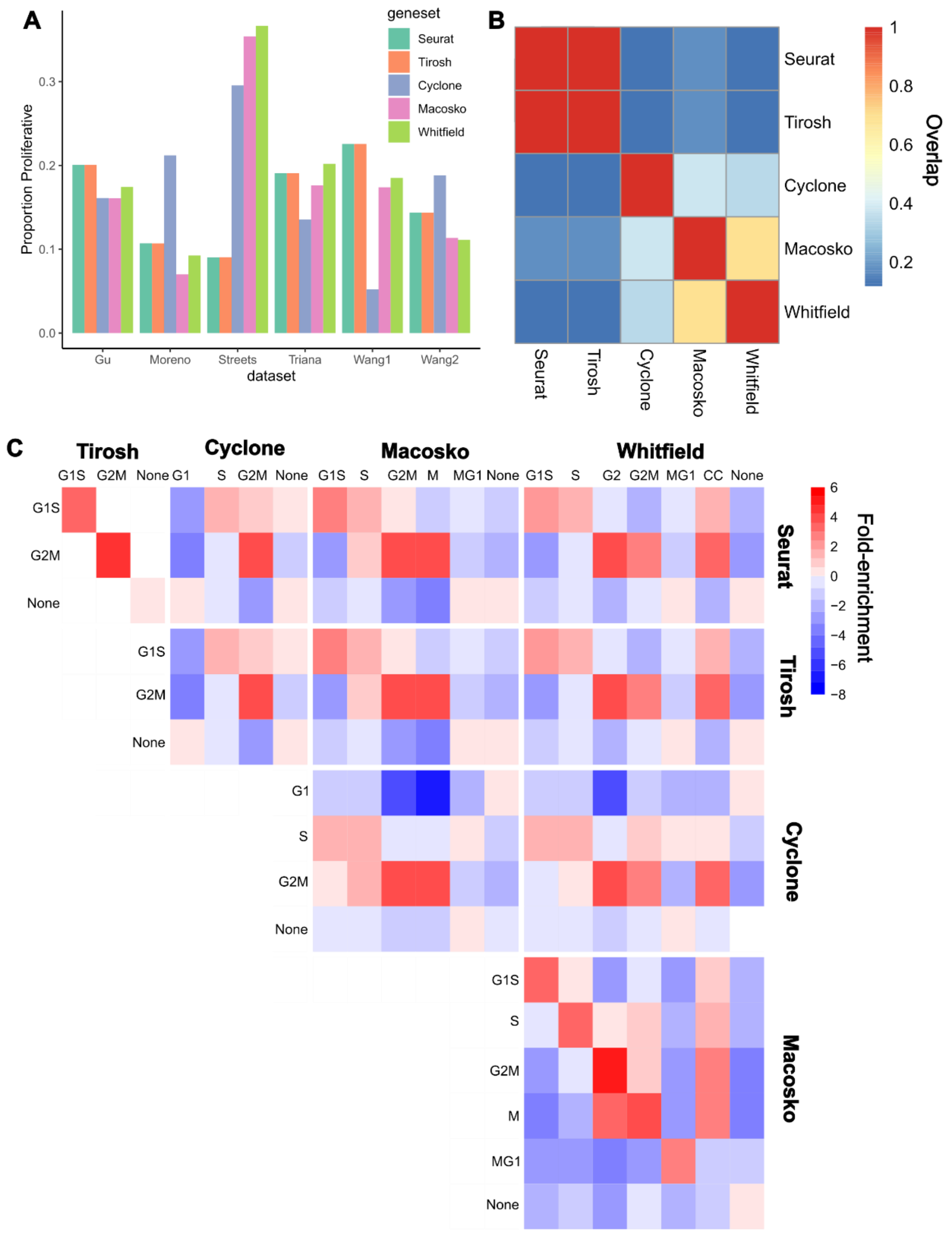

Figure S6: Effect of marker gene choice on cell-cycle classification. (A) Proportion of cells classified as proliferating (any phases other than G1, MG1, or None) in each dataset using different marker gene sets included in CycleMix. (B) Overlap of marker genes between each gene set. (C) Consistency of cell-type phase assignments over all datasets for each pair of phases identified by each mark-gene set. Consistency was calculated as fold enrichment using 2-by-2 contingency tables for each pair of cell cycle phases.

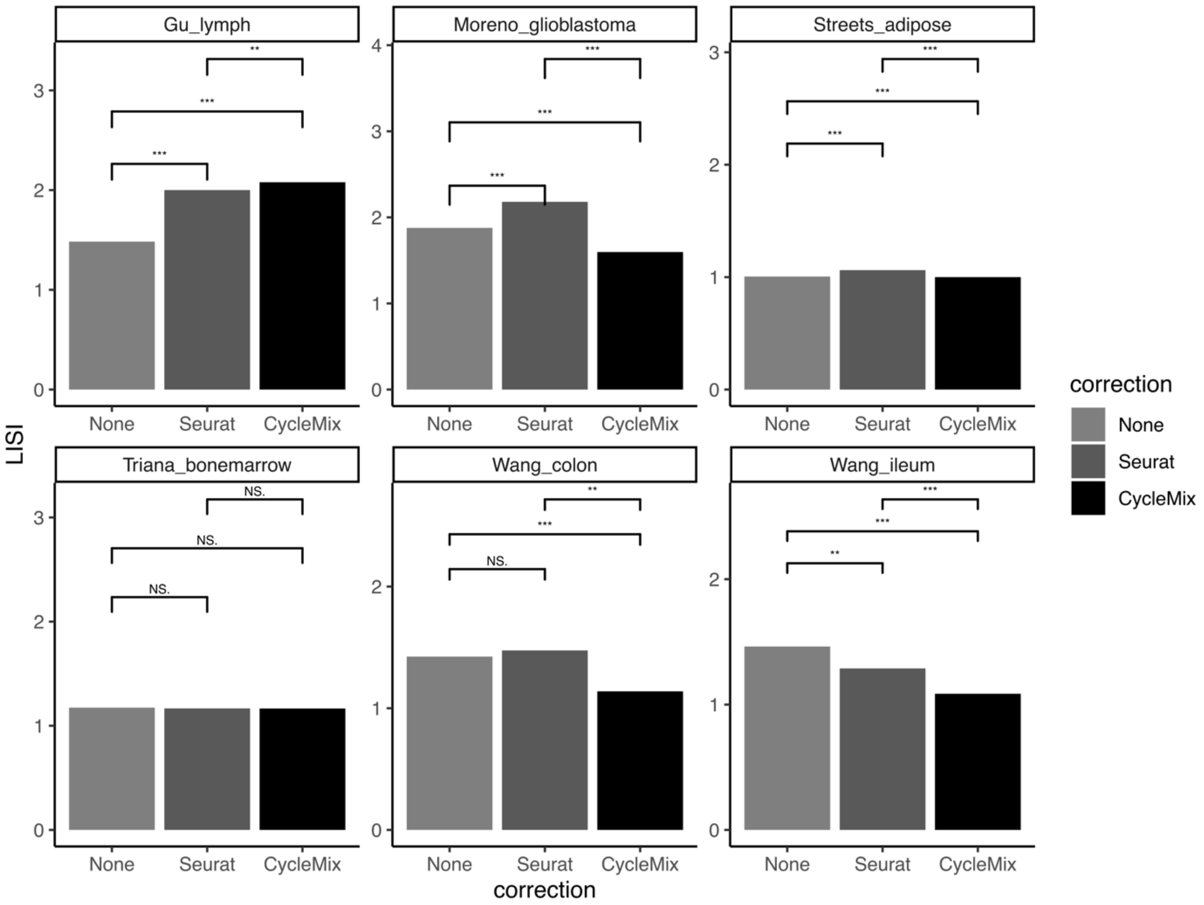

Figure S7: Average LISI scores for proliferative cell-types before and after cell-cycle correction. A LISI score of 1 (minimum possible score) indicates complete separation of the cell-types. None = prior to cell-cycle correction. Seurat = sctransform cell-cycle score regression. CycleMix = partial cell-cycle regression within proliferating cells. Significance of differences was determined using a Wilcoxon-rank-sum test. * = $p < 0.05$, ** = $p < 0.0005$, *** = $p < 5x10^{-8}$, NS. = not significant.

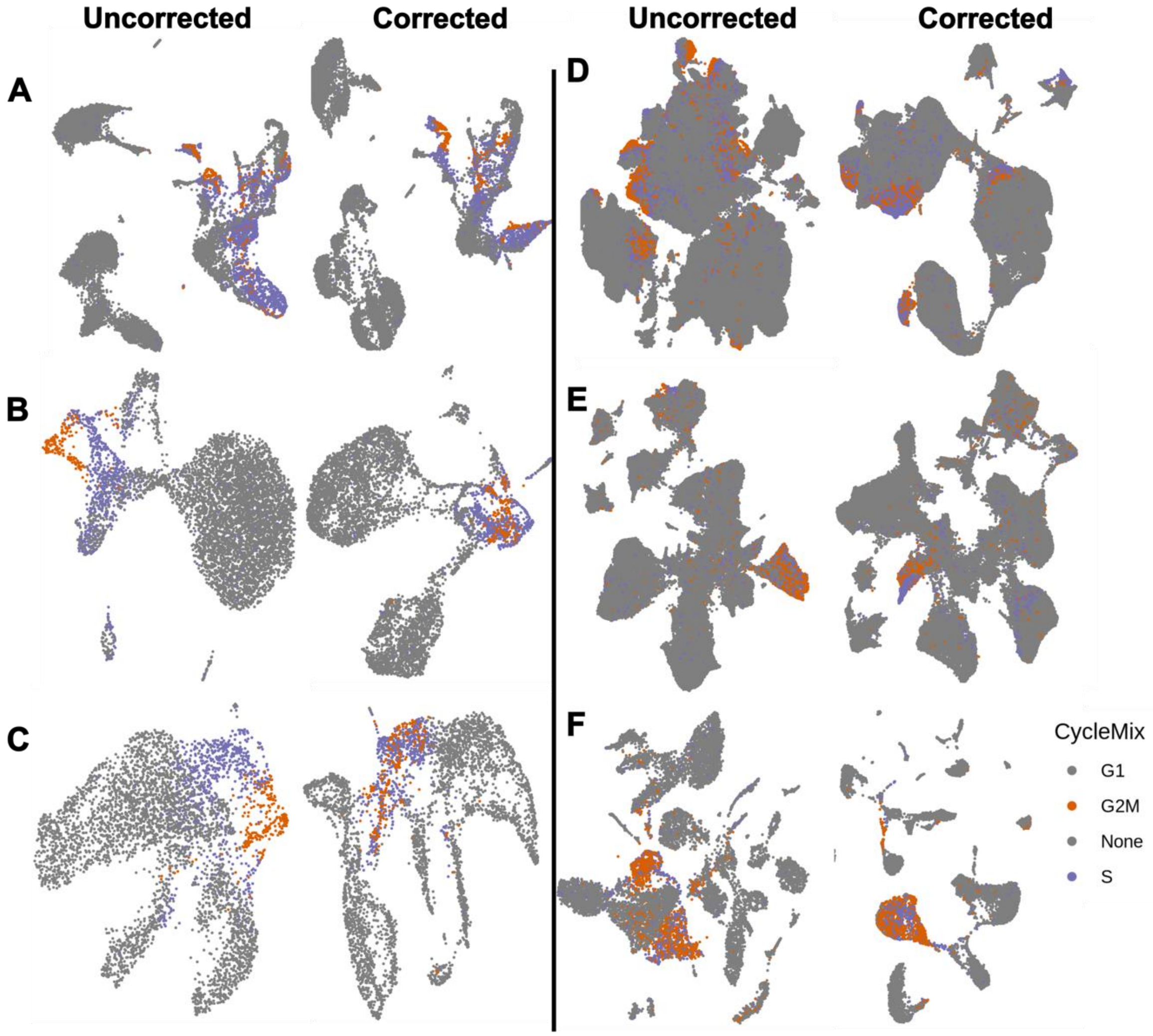

Figure S8: UMAPs for each droplet-based dataset before and after partial cell-cycle regression with CycleMix. Cells are coloured by their cell-cycle phase according to CycleMix. (A) Triana, (B) Wang ileum, (C) Wang colon, (D) Moreno, (E) Adipose, (F) Gu.

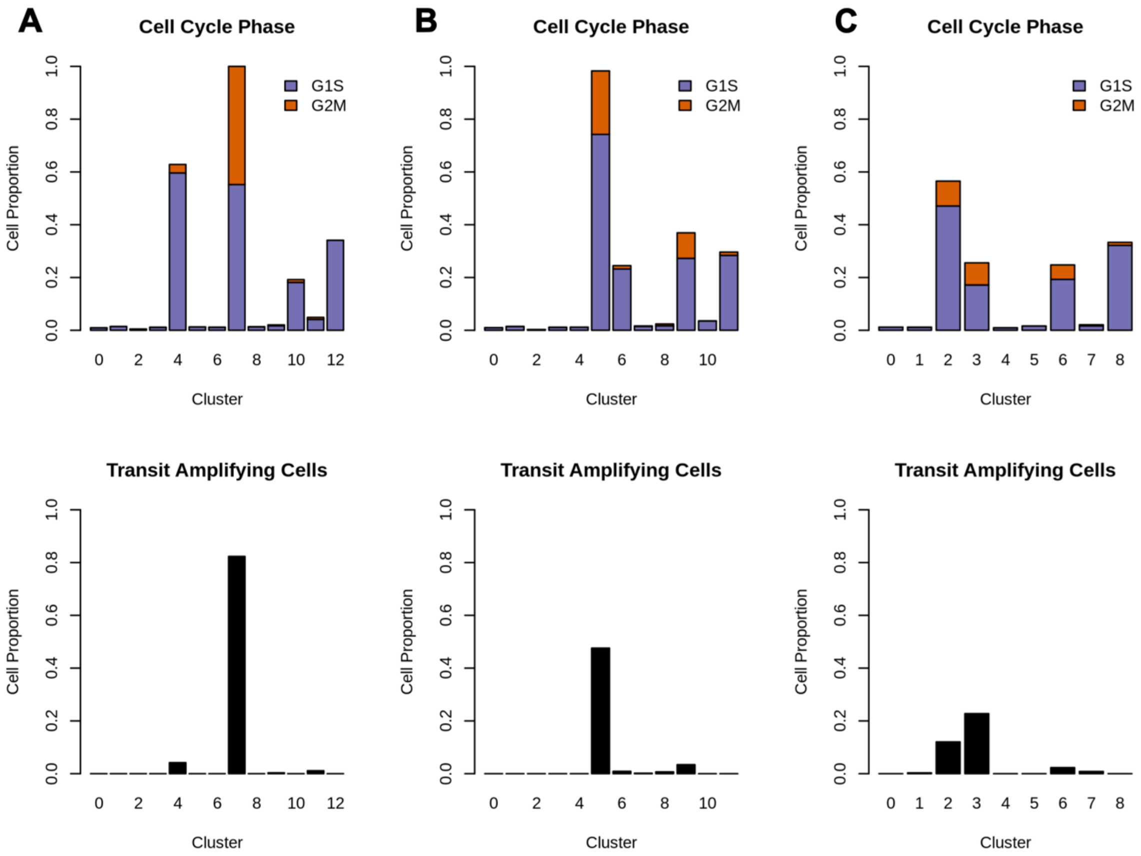

Figure S9: Cell-cycle regression of Wang Ileum. The dataset was clustered using the standard pipeline at 0.8 resolution on the uncorrected expression (A), CycleMix partial cell-cycle regression (B), and Seurat cell-cycle score regression (C). Each top plot shows the proportion of each cluster assigned to each cell-cycle phase by CycleMix. Bottom plots show the proportion of each cluster annotated as transit amplifying cells, the most proliferative cell-type in the dataset.

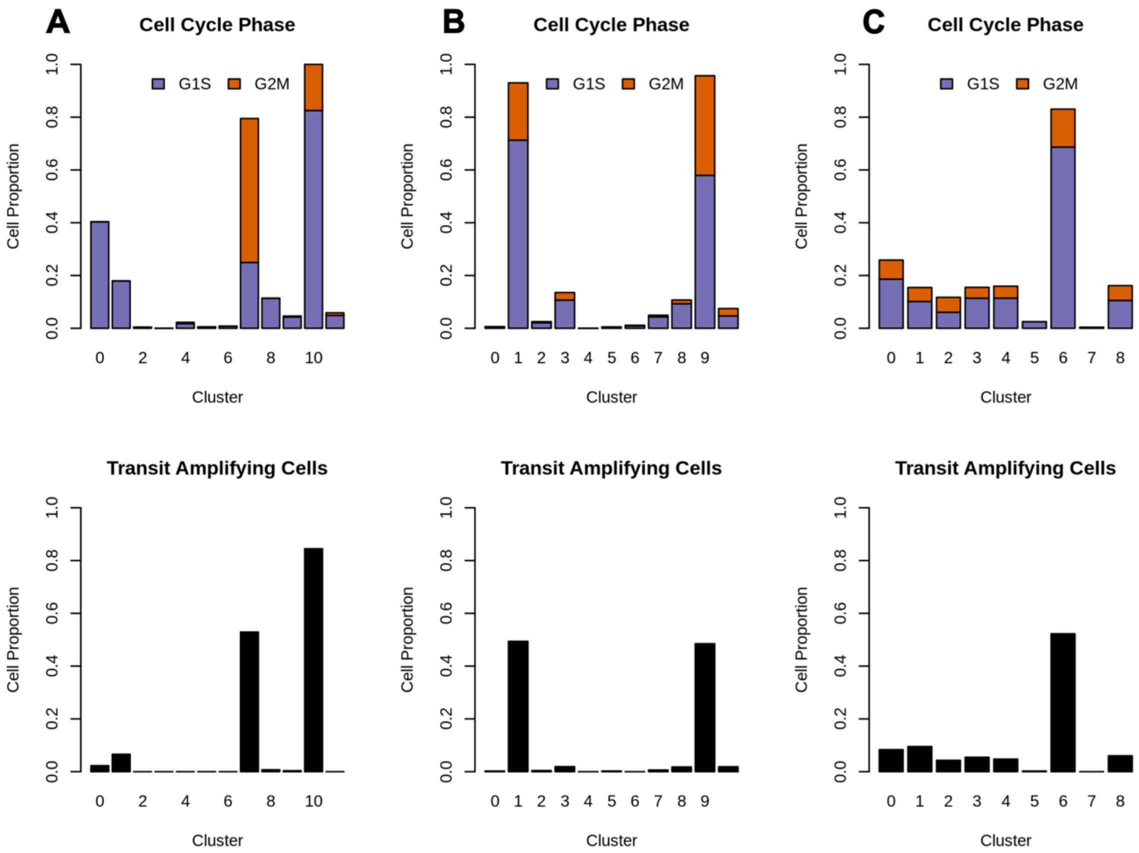

Figure S10: Cell-cycle regression of Wang Colon. The dataset was clustered using the standard pipeline at 0.8 resolution on the uncorrected expression (A), CycleMix partial cell-cycle regression (B), and Seurat cell-cycle score regression (C). Each top plot shows the proportion of each cluster assigned to each cell-cycle phase by CycleMix. Bottom plots show the proportion of each cluster annotated as transit amplifying cells, the most proliferative cell-type in the dataset.

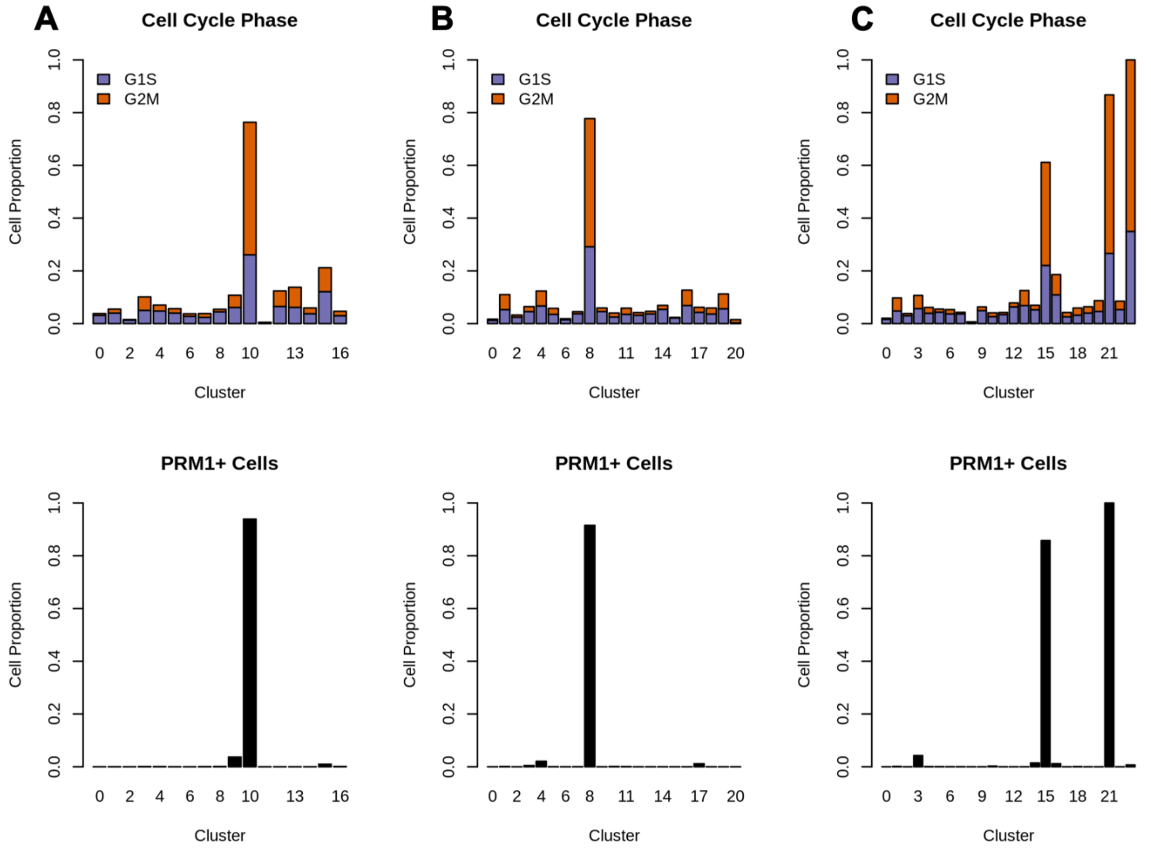

Figure S11: Cell-cycle regression of the Adipose dataset. The dataset was clustered using the standard pipeline at 0.8 resolution on the uncorrected expression (A), CycleMix partial cell-cycle regression (B), and Seurat cell-cycle score regression (C). Each top plot shows the proportion of each cluster assigned to each cell-cycle phase by CycleMix. Bottom plots show the proportion of each cluster annotated as PRM1+ cells, the most proliferative cell-type in the dataset.

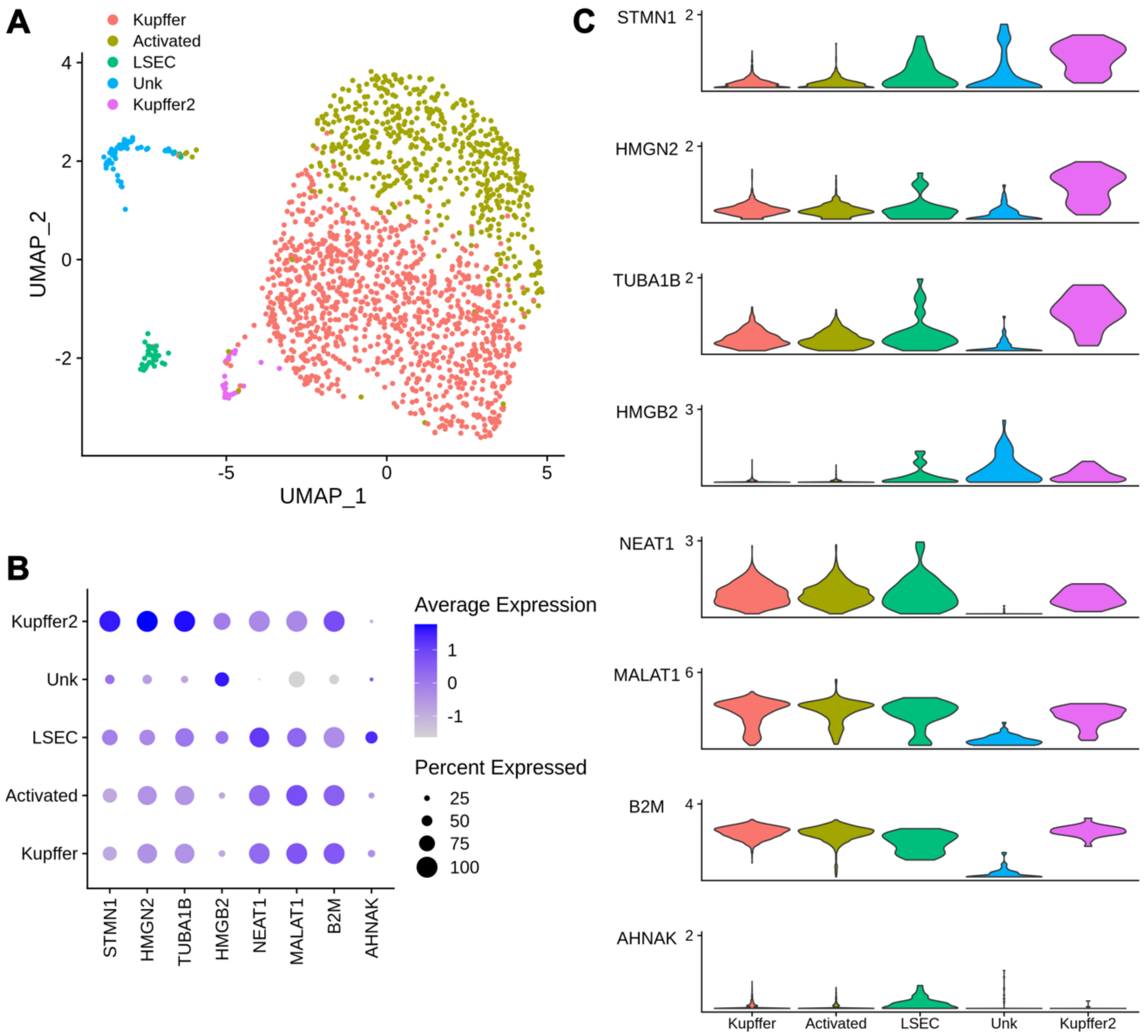

Figure S12: Cell-cycle and quiescence marker expression in iPSC-derived macrophages. (A) Annotated cell-type clusters for the iPSC-derived Kupffer cells from the original authors. The Kupffer2 cluster contains the proliferative cells. (B) Dotplot of cell-cycle marker genes (*STMN1, HMGN2, TUBA1B,* and *HMGB2*), and quiescence marker genes (*NEAT1, MALAT1, B2M, AHNAK*), aggregated at the level of cluster shown in A. (C) Violin plots of the same genes shown in B.

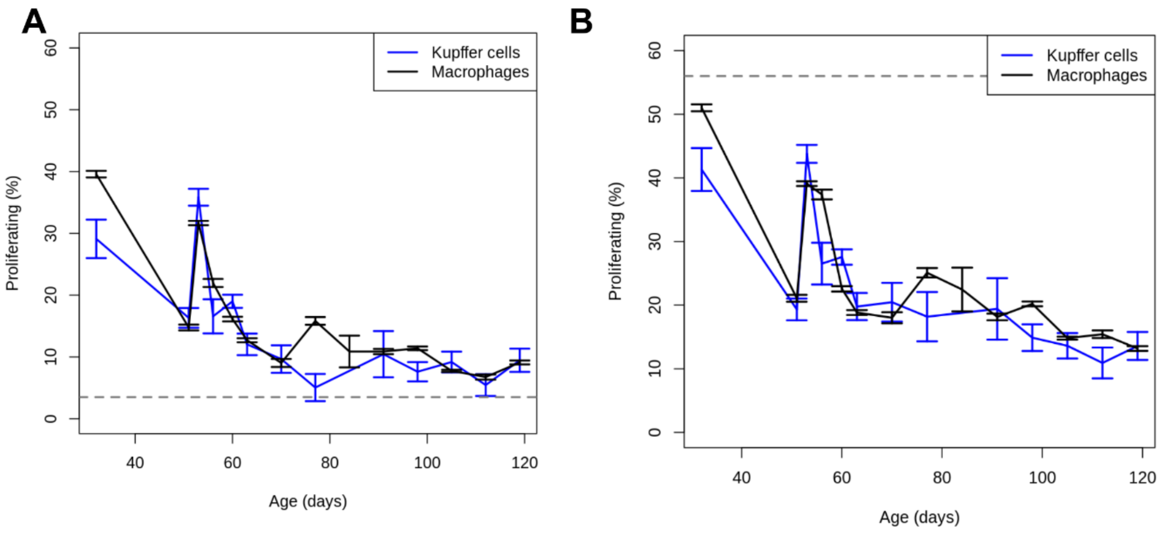

Figure S13: Proportion of fetal myeloid cells that are proliferating according to CycleMix & Seurat (A) Proportion of proliferating macrophage or Kupffer cells across human fetal development using CycleMix. Dashed line indicates proportion of iPSC-derived Kupffer cells were proliferating. Error bars indicate 95% confidence intervals. (B) the same as A only using Seurat's cell cycle classification instead.
